# IL-26 Is Overexpressed in Rheumatoid Arthritis and Induces Proinflammatory Cytokine Production and Th17 Cell Generation

**DOI:** 10.1371/journal.pbio.1001395

**Published:** 2012-09-25

**Authors:** Murielle Corvaisier, Yves Delneste, Henry Jeanvoine, Laurence Preisser, Simon Blanchard, Erwan Garo, Emmanuel Hoppe, Benjamin Barré, Maurice Audran, Béatrice Bouvard, Jean-Paul Saint-André, Pascale Jeannin

**Affiliations:** 1 LUNAM Université, Université d'Angers, Angers, France; 2 Inserm, UMR 892, Angers, France; 3 CNRS, UMR 6299, Angers, France; 4 Université d'Angers, CHU Angers, Laboratoire d'immunologie, Angers, France; 5 Université d'Angers, CHU Angers, Service de Rhumatologie, Angers, France; 6 Université d'Angers, CHU Angers, Service de Pathologie cellulaire et tissulaire, Angers, France; 7 Université d'Angers, UPRES-EA 3142, Angers, France; National Jewish Medical and Research Center/Howard Hughes Medical Institute, United States of America

## Abstract

Interleukin-26 (IL-26), a member of the IL-10 cytokine family, induces the production of proinflammatory cytokines by epithelial cells. IL-26 has been also reported overexpressed in Crohn's disease, suggesting that it may be involved in the physiopathology of chronic inflammatory disorders. Here, we have analyzed the expression and role of IL-26 in rheumatoid arthritis (RA), a chronic inflammatory disorder characterized by joint synovial inflammation. We report that the concentrations of IL-26 are higher in the serums of RA patients than of healthy subjects and dramatically elevated in RA synovial fluids compared to RA serums. Immunohistochemistry reveals that synoviolin^+^ fibroblast-like synoviocytes and CD68^+^ macrophage-like synoviocytes are the main IL-26-producing cells in RA joints. Fibroblast-like synoviocytes from RA patients constitutively produce IL-26 and this production is upregulated by IL-1-beta and IL-17A. We have therefore investigated the role of IL-26 in the inflammatory process. Results show that IL-26 induces the production of the proinflammatory cytokines IL-1-beta, IL-6, and tumor necrosis factor (TNF)-alpha by human monocytes and also upregulates the expression of numerous chemokines (mainly CCL20). Interestingly, IL-26-stimulated monocytes selectively promote the generation of RORgamma t^+^ Th17 cells, through IL-1-beta secretion by monocytes. More precisely, IL-26-stimulated monocytes switch non-Th17 committed (IL-23R^−^ or CCR6^−^ CD161^−^) CD4^+^ memory T cells into Th17 cells. Finally, synovial fluids from RA patients also induce Th17 cell generation and this effect is reduced after IL-26 depletion. These findings show that IL-26 is constitutively produced by RA synoviocytes, induces proinflammatory cytokine secretion by myeloid cells, and favors Th17 cell generation. IL-26 thereby appears as a novel proinflammatory cytokine, located upstream of the proinflammatory cascade, that may constitute a promising target to treat RA and chronic inflammatory disorders.

## Abstract 2

Human interleukin-26 induces Th17 cells, is over-expressed in rheumatoid arthritis, and is thus a promising therapeutic target in chronic inflammatory disease.

## Introduction

Rheumatoid arthritis (RA), the most common form of chronic inflammatory arthritis, is characterized by persistent synovial inflammation, systemic inflammation, and autoantibodies [1]. The multiple proinflammatory cascades described in RA lead to persistent synovitis, resulting in articular cartilage and bone damages [1]. The proinflammatory cytokines tumor necrosis factor (TNF)-alpha, interleudin (IL)-1-beta, and IL-6, produced by synovial cells and infiltrating cells, actively participate to synovitis and joint destruction [1],[2]. Although RA has been first considered as a Th1-mediated disease, the proinflammatory Th17 cells (the major source of IL-17A; reviewed in [3]) have been recently reported in RA [4]–[6], mainly in early and non-treated RA [7]–[9]. IL-17A induces proinflammatory cytokine and chemokine secretion by synovial fibroblasts, macrophages, chondrocytes, and osteoblasts, and participates in tissue remodeling by inducing the production of growth factors, matrix metalloproteinases, and RANK ligand [5],[10]. In vivo, the severity of collagen- or adjuvant-induced arthritis is reduced with IL-17A deficiency or blockade (using antibodies or a receptor antagonist) (reviewed in [4],[11]).

Besides disease-modifying antirheumatic and anti-inflammatory drugs, TNF-alpha inhibitors have been proven to be effective in RA [1]. However, some patients fail to respond to TNF-alpha inhibitors, present short-term responses or adverse effects [1]. Currently, an increasing number of cytokine inhibitors, such as anti-IL-17A antibodies, are under investigation in RA treatment [2],[10],[11]. As early treatment preserves joint function, factors involved in the early phase of the inflammatory cascade and/or in Th17 cell generation constitute preferred therapeutic targets.

IL-26, also known as AK155, is a member of the IL-10 cytokine family that includes IL-10, interferon (IFN)-λs (IL-28A/B and IL-29), and the IL-20 subfamily (IL-19, IL-20, IL-22, IL-24, and IL-26) [12],[13]. Although these cytokines show strikingly similar secondary structures, IL-26 shares very low sequence homology (∼15% to 25%) with other members of the IL-20 subfamily [13],[14]. IL-26 is a 19-kDa α-helical protein that forms stable homodimers and presents a predicted isoelectric point of 10.7 [15],[16]. The *il-26* gene is conserved in most vertebrate species (orthologs of the *il-26* gene have been identified in several non-mammalian species) but absent in most rodent strains (including mice and rat) [14],[17]. IL-26 was first described as a gene whose expression is upregulated in herpesvirus saimiri-transformed T cells [15]. The expression of IL-26 is restricted to some T cell and natural killer (NK) cell subsets [18]–[20]. The protein IL-26 has been evidenced in some Th17 cells infiltrating colonic lesions in patients with Crohn's disease [21]. Some Th17 cells isolated from psoriasis patients, and, to a lesser extent, Th1 cells, but not Th2 and regulatory T cells, express IL-26 mRNA [18],[22],[23]. Upon stimulation, NK cells also express IL-26 mRNA [19]. Stage 3 immature CD117^+^ CD161^+^ NK cells (present in secondary lymphoid tissues) and CD56^+^ NKp44^+^ NK-22 cells (located in mucosa-associated lymphoid tissues) express higher levels of IL-26 mRNA than other NK cell subsets (stage 4 and NKp44^−^ NK cells) [24],[25].

IL-26 has been reported to signal via the IL-10R2/IL-20R1 heterodimeric receptor [16],[26]. While IL-10R2 is broadly expressed, IL-20R1 is expressed by many epithelial cell types, but not by hematopoietic cells [18]–[21]. The only biological activity of IL-26 reported so far is the upregulation of IL-8, IL-10, TNF-alpha, and/or CD54 expression by intestinal epithelial cell lines, associated to a phosphorylation of STAT3 (and/or STAT1) [16],[21].

Even though an upregulation of IL-26 has been reported in Crohn's disease, a Th1- and Th17-mediated inflammatory disorder, the potential role of IL-26 in human disease remains partially unknown [21].

We have investigated in this study the expression and role of IL-26 in RA. We report that RA patients exhibit high concentrations of IL-26 in serums and synovial fluids (SFs) and that RA synoviocytes constitutively produce IL-26. We also show that IL-26 triggers the production of proinflammatory cytokines by monocytes that induce non-Th17 memory T cell differentiation into Th17 cells. IL-26 may therefore represent a novel therapeutic target in RA and chronic inflammatory disorders.

## Results

### IL-26 Is Overexpressed in Rheumatoid Arthritis Patients

**Figure 1:**
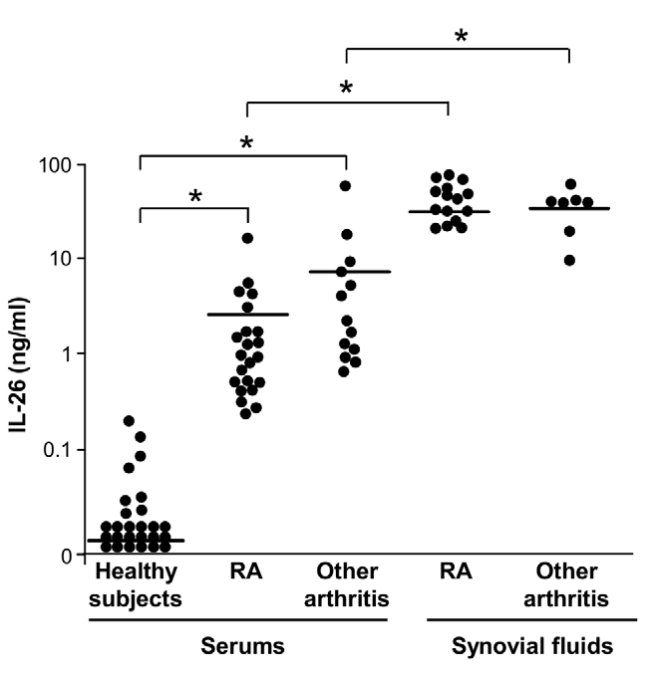
IL-26 concentrations in the serums and SFs of RA patients. IL-26 was quantified by ELISA in the serums of 26 healthy subjects, 22 RA patients, and 13 patients with other inflammatory arthritis (spondylarthritis, psoriatic arthritis, rhizomelic polyarthritis, and undifferentiated inflammatory arthritis) and in the SFs of 15 RA patients and seven patients with other inflammatory arthritis. Lines correspond to the mean values; * *p*<0.05 (Mann Whitney test).

We quantified IL-26 by ELISA in the serums of RA patients and healthy subjects. IL-26 concentrations were higher in RA patients (2.43±3.80 ng/ml; mean ± standard deviation [SD], *n* = 22) than in healthy subjects (0.03±0.04 ng/ml; *n* = 26) (Figure 1). As RA is characterized by synovial inflammation, IL-26 was also quantified in RA SF. IL-26 concentrations were higher in RA SF (46.63±21.92 ng/ml; *n* = 15) than in RA serums (Figure 1). IL-26 concentrations correlated with IL-1-beta concentrations in RA serums (*r* = 0.95, *p*≤10^−4^) and RA SF (*r* = 0.6, *p*≤0.03) (Figure S1) but not with C-reactive protein (CRP), IL-6, TNF-alpha, and total leukocyte counts (unpublished data). Elevated concentrations of IL-26 were also detected in the serums (8.82±16.32 ng/ml; mean ± SD, *n* = 13) and SF (55.19±29.24; *n* = 7) of patients with other inflammatory arthritis (Figure 1) and were associated to a local inflammation, as evidenced by high levels of IL-1beta and TNF-alpha in the SF (unpublished data). The levels of IL-26 in RA serums and SF were unaffected by rheumatoid factor depletion, allowing excluding interference in IL-26 quantification (unpublished data).

These results demonstrate an overexpression of IL-26 in the serums and SF of patients with RA and other inflammatory arthritis.

### Synoviocytes Are a Major Source of IL-26 in RA Synovium

**Figure 2:**
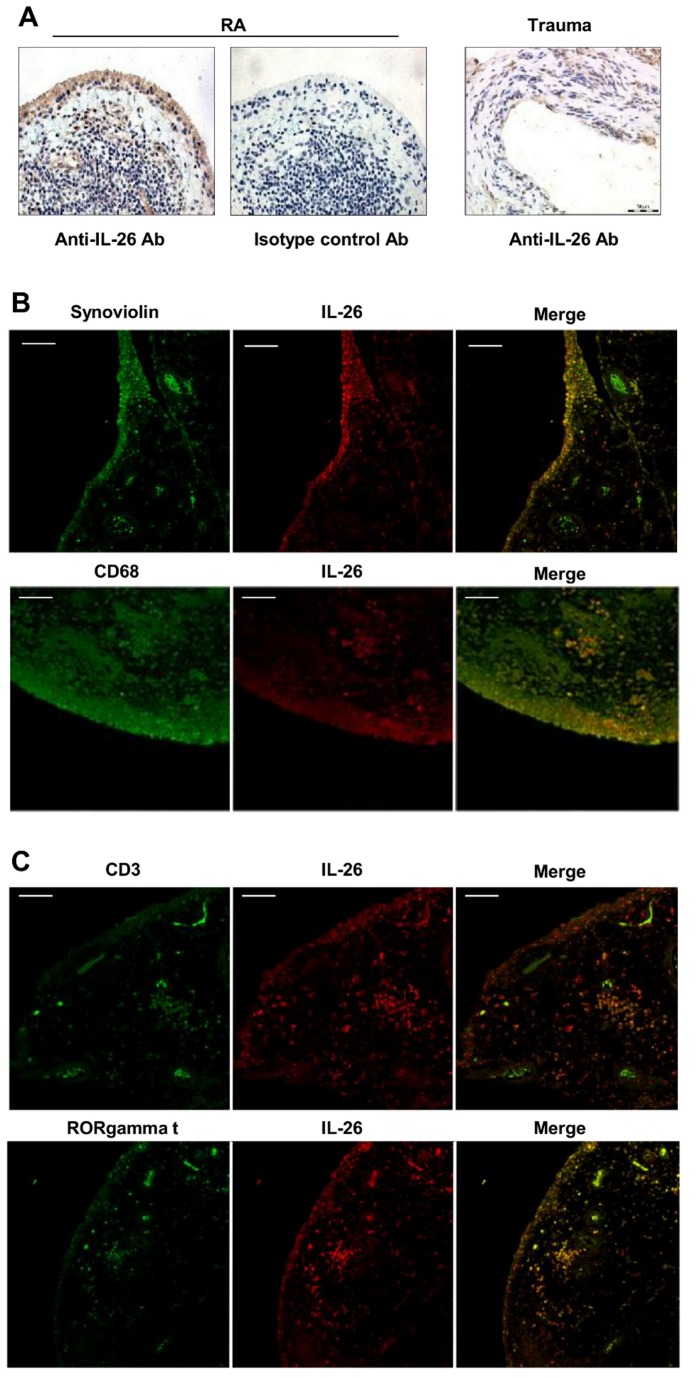
Immunohistological analysis of IL-26 expression in RA synovium. (A) IL-26 expression was analyzed in the synovium of RA patients or trauma patients by immunohistochemistry using an anti-IL-26 mAb (original magnification: ×20). An IgG2b mAb was used as control. Pictures are representative of the results obtained with three RA patients and two patients with recurrent dislocation. (B and C) Immunofluorescence in RA synovium using a biotinylated anti-IL-26 mAb (red) and antibodies directed either against the cell lineage markers (green) CD68 and synoviolin (B), CD3 or RORgamma (C); bars = 100 µm. Pictures are representative of the results obtained with tissues from three RA patients.

**Figure 3:**
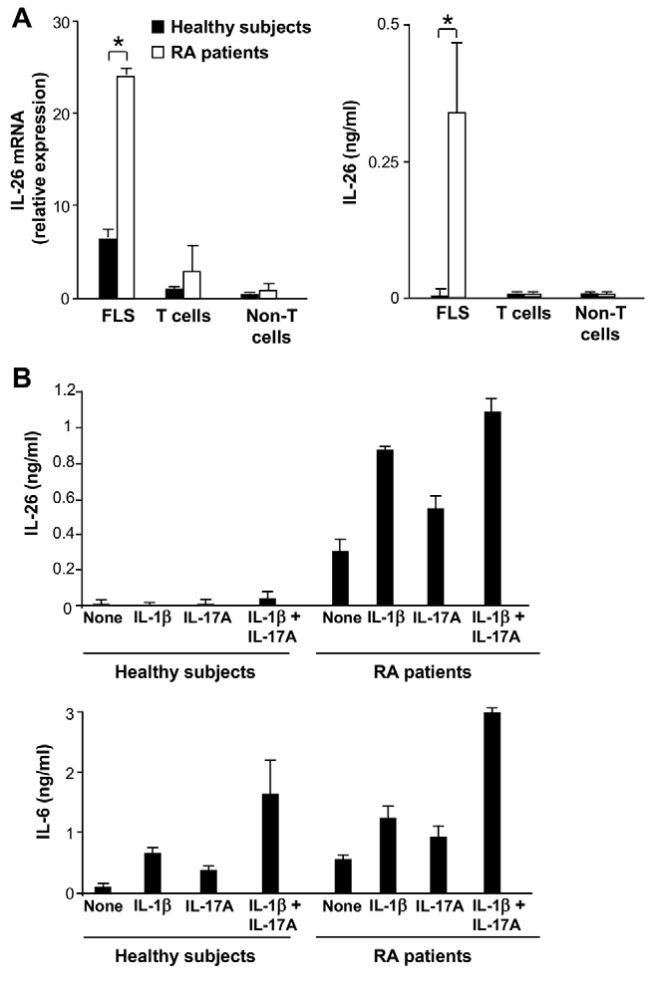
Synovial fibroblasts are the main IL-26-producing cells in RA joints. (A) FLS from RA patients and healthy subjects (*n* = 3), and T cells and non-T mononuclear cells (isolated from RA SF cells and from PBMC of healthy subjects) (*n* = 3), were either unstimulated or stimulated with anti-CD3 plus -CD28 mAbs or soluble CD40L, respectively. IL-26 mRNA expression (expressed as relative mRNA expression) was analyzed by RT-qPCR after 6 h stimulation (left panel) and IL-26 production (in ng/ml) was determined by ELISA after 48 h stimulation (right panel). Results are expressed as mean ± SD, *n* = 3; *p<0.05 (Wilcoxon matched-pairs signed-ranks test). (B) IL-26 (upper panel) and IL-6 (lower panel) were quantified by ELISA in the supernatants of FLS from healthy subjects and RA patients, stimulated or not with 50 ng/ml IL-1-beta and/or IL-17A. Results are expressed in ng/ml (mean ± SD, *n* = 3).

Higher IL-26 concentrations in SF than in serums might suggest a local production of IL-26 in RA synovial tissues. Immunohistochemical analysis revealed a strong expression of IL-26 in the hyperplastic lining cell layer of RA synovium (Figure 2A, left panel). In contrast, IL-26 was poorly expressed in the synovium of trauma patients (Figure 2A, right panel). No staining was observed with a control IgG2b monoclonal antibody (mAb) in RA (Figure 2A, middle panel) and in trauma synovium (unpublished data). Double immunofluorescence staining of RA synovium revealed that synoviolin^+^ fibroblast-like synoviocytes (FLSs) (Figure 2B, upper panel) and CD68^+^ macrophage-like synoviocytes, located in the hyperplastic lining cell layer (Figure 2B, lower panel), expressed IL-26. In support, primary FLS from RA synovial tissues expressed higher levels of IL-26 mRNA than FLS from healthy subjects (Figure 3A, left panel), and constitutively produced IL-26, in contrast to FLS from healthy subjects (Figure 3A, right panel). IL-26 production by RA FLS was upregulated by IL-1-beta and IL-17A (Figure 3B, upper panel), two cytokines involved in RA pathogenesis [2],[5]. As previously reported [27],[28], FLS from healthy subjects produced IL-6 (Figure 3B, lower panel) but low or undetectable IL-26 (Figure 3B, upper panel) in response to a stimulation with IL-1-beta, IL-17A, or IL-1-beta plus IL-17A. Interestingly, some CD3^+^ T cells, including RORgamma t^+^ T cells, located in lymphocyte aggregates in the sublining layers, also expressed IL-26 (Figure 2C).

Among the mononuclear cells isolated from RA SF, a low expression of IL-26 mRNA was detected in stimulated T cells but not in non-T cells (Fig. 3A, left panel). No IL-26 was detected in the supernatants of these two cell populations (Figure 3A, right panel). T cells and non-T cells isolated from the blood of healthy subjects, either stimulated or not, expressed low levels of IL-26 mRNA and did not produce detectable levels of IL-26 (Figure 3A). Finally, IL-26 secretion and mRNA expression was also undetectable in neutrophils purified from the SFs of RA patients (unpublished data).

These results identify RA synoviocytes as a major source of IL-26 in inflamed joints of which the production is upregulated by IL-1-beta and IL-17A.

### IL-26 Upregulates Proinflammatory Cytokine Expression by Myeloid Cells

**Figure 4:**
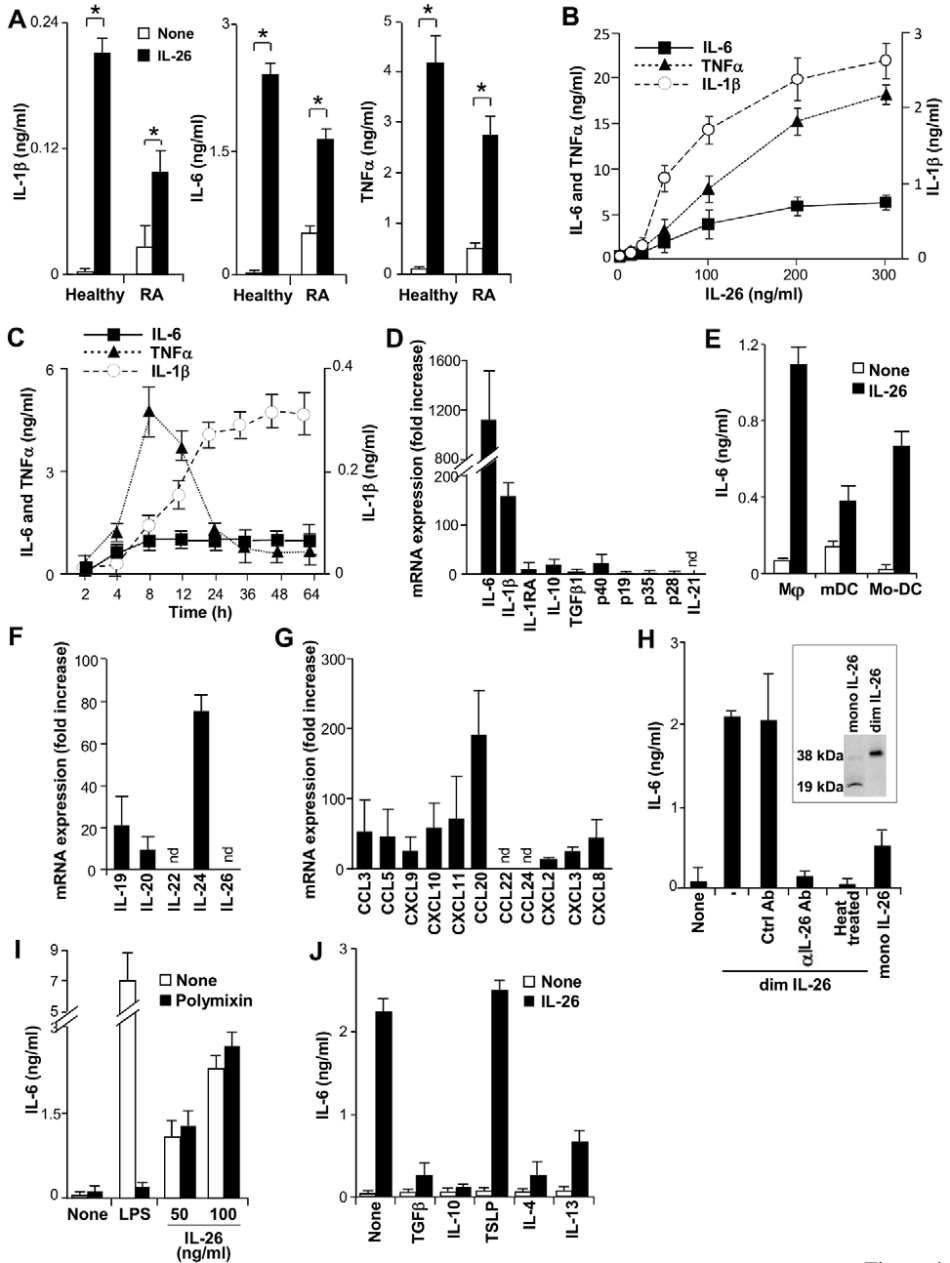
IL-26 upregulates proinflammatory cytokine secretion by myeloid cells. (A) IL-1-beta, IL-6, and TNF-alpha were quantified by ELISA in the 16 h (IL-6 and TNF-alpha) or 48 h (IL-1-beta) supernatants of monocytes isolated from the blood of healthy subjects or CD14^+^ myeloid cells isolated from RA SF cells and exposed or not to 50 ng/ml IL-26. (B) Monocytes were exposed to increasing concentrations of IL-26. IL-1-beta, IL-6, and TNF-alpha were quantified by ELISA in the 16 h (IL-6 and TNF-alpha) or 48 h (IL-1-beta) supernatants. (C) Monocytes were exposed to 50 ng/ml IL-26 and IL-1-beta, IL-6, and TNF-alpha were quantified by ELISA in the supernatants collected at the indicated time-points. (D) Monocytes were exposed to 50 ng/ml IL-26 for 6 h and the expression of the mRNA encoding the indicated cytokines was analyzed by RT-qPCR. Results are expressed in fold increase of mRNA expression compared to unstimulated cells (mean ± SD, *n* = 3); nd, means not detectable. TNF-alpha mRNA expression, measured after 2 h stimulation [55], was increased of 15 fold (unpublished data). (E) Monocyte-derived macrophages (MΦ), BDCA1^+^ peripheral blood myeloid dendritic cells (mDC), and monocyte-derived DC (Mo-DC) were cultured without or with 50 ng/ml dimeric IL-26. IL-6 was quantified by ELISA in the 16 h supernatants. (F–G), Monocytes were exposed for 6 h to 50 ng/ml dimeric IL-26 (dim IL-26) and the mRNA encoding members of the IL-10 cytokine family (F) and chemokines (G) were analyzed by RT-qPCR. Results are expressed in fold increase of mRNA expression, compared to unstimulated cells. (H) Monocytes were cultured in the presence of 50 ng/ml IL-26, 50 ng/ml IL-26 plus 10 µg/ml neutralizing goat anti-IL-26 Ab or a control Ab, 50 ng/ml heat-treated IL-26, or 50 ng/ml monomeric IL-26 (mono IL-26). Insert, Western-blotting analysis of monomeric and dimeric IL-26. (I) Monocytes were cultured in the presence of 50 or 100 ng/ml IL-26 or 100 pg/ml LPS, with or without 0.2 µg/ml polymixin B. (J) Monocytes were cultured in the presence or absence of 50 ng/ml IL-26, with or without 20 ng/ml IL-4, IL-10, IL-13, TGF-beta, or TSLP. (H–J) IL-6 was quantified in the 16 h supernatants. (A–C, E, H–J) Results are expressed in ng/ml (mean ± SD, *n* = 5). * *p*<0.05 (Wilcoxon matched-pairs signed-ranks test).

As myeloid cells control the inflammatory process and the polarization of T cells, we investigated their sensitivity to IL-26. CD14^+^ myeloid cells isolated from RA SF and monocytes isolated from the blood of healthy subjects were incubated with homodimeric IL-26 (hereafter referred to as IL-26) in serum-free medium. IL-26 induced IL-1-beta, IL-6, and TNF-alpha production by monocytes from healthy subjects and upregulated their secretion by myeloid cells from RA SF (Fig. 4A). This effect was dose- (Figure 4B) and time-dependent (Figure 4C), and associated to an increase of expression of the corresponding mRNA (unpublished data) (Figure 4D). Among the IL-20 cytokine subfamily members (IL-19, IL-20, IL-22, and IL-24), IL-26 was the only that induced proinflammatory cytokine secretion by monocytes (unpublished data). Monocyte-derived macrophages (MΦ), peripheral blood myeloid BDCA1^+^ DC (mDC), and monocyte-derived dendritic cells (Mo-DC) were also sensitive to IL-26, as evidenced by an increase of IL-6 production (Figure 4E). IL-26 also upregulated the expression of the mRNA encoding IL-19, IL-20, and IL-24 by monocytes (Figure 4F), three members of the IL-10 family of which the expression is upregulated in RA [12]. IL-26 also induced a huge increase of the mRNA encoding CC-chemokine ligand 20 (CCL20) (involved in Th17 cell recruitment), and, to a lesser extent, of the mRNA encoding CCL3, CCL5, CXCL9, CXCL10, and CXCL11 (involved in Th1 cell recruitment), CXCL2, CXCL3, and CXCL8 (involved in neutrophil recruitment), while the expression of the mRNA encoding CCL22 and CCL24 (involved in Th2 cell recruitment) was unaffected (Figure 4G). IL-26 did not modulate the production (unpublished data) and poorly affected the expression of the mRNA encoding the immunomodulatory molecules IL-10, TGF-beta, IL-27 (p28/EBI3), and IL-1 receptor antagonist (IL-1RA), and the immunostimulatory cytokines IL-12 (p40/p35) and IL-23 (p40/p19) (Figure 4D). As expected, IL-17A, IL-21, IL-22, and IL-26 mRNA expression remained undetectable in monocytes, whatever the stimulus used (unpublished data) (Figure 4F).

We also observed that dimeric IL-26 from eBiosciences and R&D Systems gave similar results (unpublished data) and that monomeric IL-26 was less potent than dimeric IL-26 in activating monocytes (Figure 4H). As monocytes are highly sensitive to endotoxin, we excluded a potential role of contaminating endotoxin. First, endotoxin concentrations in monomeric and dimeric recombinant IL-26 were <1.0 EU per µg of protein using the limulus amebocyte lysate (LAL) assay (data from manufacturers) and <10 pg lipopolysaccharide (LPS) per µg of protein using an in vitro cell-based LPS detection assay (Cayla-Invivogen) (unpublished data). Second, IL-26-induced IL-6 production by monocytes was unaffected by polymyxin B, in contrast to the production of IL-6 induced by LPS from the *Escherichia coli* strain K12 (Figure 4I). Third, the IL-26-induced IL-6 production was abolished by a neutralizing anti-IL-26 Ab (but not by a control Ab) and by heat treatment (Figure 4H).

We also evaluated whether some cytokines may modulate IL-26-induced monocyte activation. Among the cytokines tested (IL-2, IL-3, IL-4, IL-5, IL-7, IL-8, IL-9, IL-10, IL-11, IL-12, IL-13, IL-15, IL-17A, IL-19, IL-20, IL-21, IL-22, IL-23, IL-24, IL-26, IL-28A, IL-29, IL-31, IFN-alpha, IFN-beta, IFN-gamma, M-CSF, TGF-beta, LIF, OSM, CT-1, and TSLP), only IL-4, IL-10, IL-13, and TGF-beta prevented IL-26-induced IL-6 production (Figure 4J).

Collectively, these results demonstrate that IL-26 induces the expression of proinflammatory cytokines by myeloid cells.

### IL-26 Selectively Promotes Th17 Cell Generation

**Figure 5:**
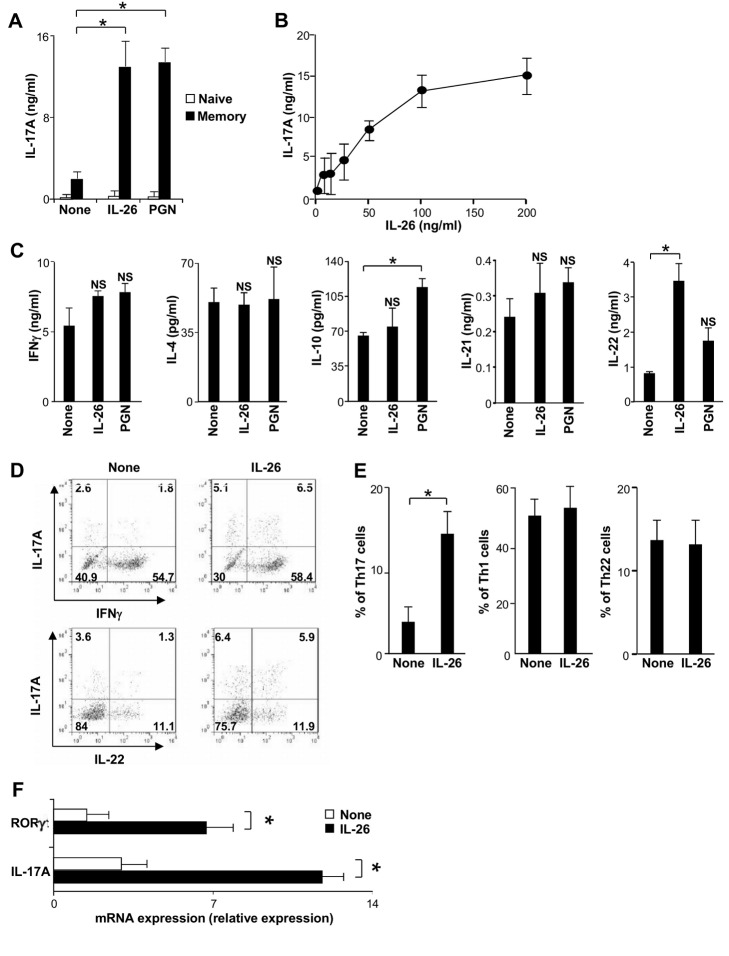
IL-26 upregulates IL-17A secretion by memory CD4^+^ T cells. (A) Naive or memory CD4^+^ T cells were stimulated by an anti-CD3 mAb, in the presence of monocytes, with or without 50 ng/ml IL-26 or 5 µg/ml PGN. IL-17A was quantified by ELISA in the 7-d supernatants. (B) Memory CD4^+^ T cells were stimulated by an anti-CD3 mAb, in the presence of monocytes, with or without IL-26 at the indicated concentrations. IL-17A was quantified in the 7-d supernatants. (C) Memory CD4^+^ T cells were stimulated as described, with or without 50 ng/ml IL-26. IFN-gamma, IL-4, IL-10, IL-21, and IL-22 were quantified by ELISA in the 7-d supernatants. (A–C) Results are expressed in ng/ml or pg/ml (mean ± SD, *n* = 5). (D and E) Memory CD4^+^ T cells, stimulated for 7 d by an anti-CD3 mAb in the presence of monocytes, with or without 50 ng/ml IL-26, were further cultured for 7 d. Then, cells were stimulated by PMA plus ionomycin and intracellular expression of IL-17A, IL-22, and IFNγ was analyzed by flow cytometry. (D) Dot plots are representative of one out of five experiments; (E) Results are expressed as a percentage of Th17- (IL-17A^+^), Th1- (IFN-gamma^+^ IL-17A^−^), or Th22- (IL-22^+^ IL-17A^−^) expressing cells (mean ± SD, *n* = 4). (F) Memory CD4^+^ T cells were stimulated by an anti-CD3 mAb in the presence of monocytes, with or without 50 ng/ml IL-26. At day 7, T cells were purified and IL-17A and RORgamma t mRNA expression was analyzed by RT-qPCR. Results are expressed as a relative mRNA expression level (mean ± SD, *n* = 4). *p<0.05 (Wilcoxon matched-pairs signed-ranks test).

RA is characterized by excessive proinflammatory Th1 and Th17 responses [4],[5]. We therefore evaluated whether IL-26-treated monocytes may modulate CD4^+^ T cell polarization. Highly purified naive and memory human CD4^+^ T cells were cultured with autologous monocytes and an anti-CD3 mAb, in the presence or absence of IL-26. IFN-gamma, IL-4, IL-10, IL-17A, IL-21, and IL-22 production was evaluated to monitor Th1, Th2, regulatory T cells (Tr1), Th17, follicular helper T cells (Tfh), and Th22 cells, respectively [29]. IL-26 upregulated IL-17A secretion by memory T cells (Figure 5A), in a dose-dependent manner (Figure 5B), and in a similar extent to peptidoglycan (PGN) (Figure 5A), used as a positive control [30]. IL-26 also upregulated IL-22 secretion by memory T cells, while IFN-gamma, IL-4, IL-10, and IL-21 production was unaffected (Figure 5C). IL-26 had no effect on the production of these cytokines by naive CD4^+^ T cells (unpublished data) (Figure 5A).

We then analyzed by fluorescence-activated cell sorting (FACS) the frequency of Th17 and/or Th22 cells in memory CD4^+^ T cells stimulated with an anti-CD3 mAb, in the presence of monocytes and IL-26. IL-26 enhanced the frequency of IL-17A-producing T cells (3.9±1.9% and 14.2±2.8%, without or with IL-26, respectively; mean ± SD, *n* = 4), expressing or not IFN-gamma or IL-22 (Figure 5D and 5E). The percentage of Th1 (IFN-gamma^+^ IL-17A^−^) and Th22 cells (IL-22^+^ IL-17^−^) was unaffected (Figure 5E). Supporting these observations, IL-26 increased the expression of the mRNA encoding the Th17-associated molecules IL-17A and RORgamma t^+^ in memory CD4^+^ T cells (Figure 5F).

These results show that, in the presence of monocytes, IL-26 selectively enhances the frequency of Th17-polarized CD4^+^ memory T cells.

### IL-26 Favors Th17 Cell Generation through IL-1-Beta Secretion by Monocytes

**Figure 6:**
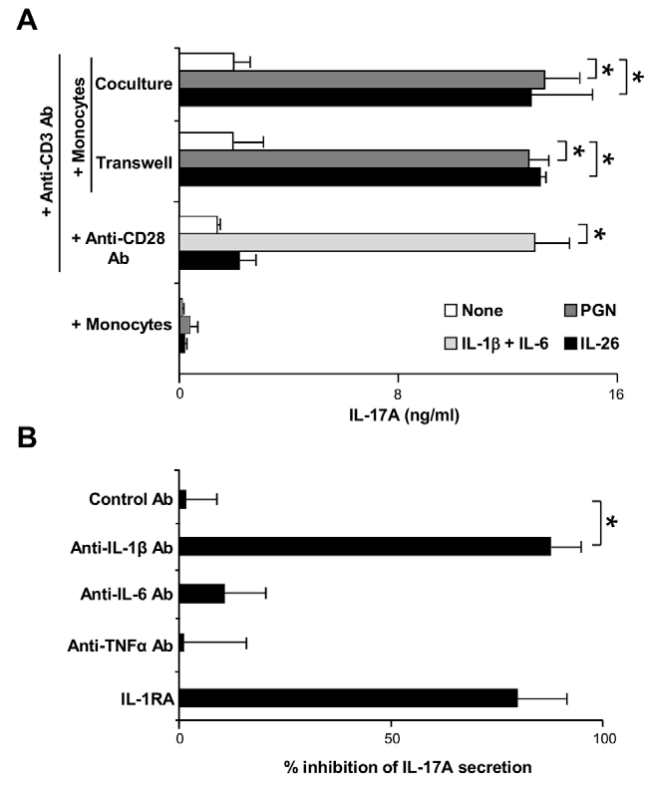
IL-26 promotes IL-17A production by memory CD4^+^ T cells through IL-1-beta secretion by monocytes. (A) Memory CD4^+^ T cells were stimulated with an anti-CD3 Ab, with or without 50 ng/ml IL-26, in the presence of (i) monocytes, (ii) monocytes cultured in separate chambers (Transwell inserts), or (iii) an anti-CD28 Ab. Memory CD4^+^ T cells were also cultured with monocytes in the absence of anti-CD3 or anti-CD28 Ab. PGN (5 µg/ml) or 10 ng/ml IL-1-beta plus 50 ng/ml IL-6 were used as positive controls. After 1 wk, IL-17A was quantified by ELISA in the supernatants. Results are expressed in ng/ml (mean ± SD, *n* = 4). (B) Memory CD4^+^ T cells were stimulated by an anti-CD3 Ab plus monocytes, with or without 50 ng/ml IL-26, in the presence or absence of 10 µg/ml neutralizing anti-IL-1-beta, anti-IL-6, anti-TNF-alpha, isotype control Abs, or 1 µg/ml soluble IL-1RA. IL-17A was quantified after 1 wk. Results are expressed as percentage of inhibition of IL-17A secretion (mean ± SD, *n* = 4). (A and B) *p<0.05 (Wilcoxon matched-pairs signed-ranks test).

We then investigated the mechanism(s) involved in the generation of Th17 cells induced by IL-26. In the absence of monocytes, IL-26 did not upregulate IL-17A production by memory CD4^+^ T cells, in contrast to IL-1-beta and IL-6 (Figure 6A), used as a positive control [30], showing that the presence of monocytes is required. IL-26 still upregulated IL-17A production when T cells were cultured (i) with monocytes in separate chambers (Transwell assay) (Figure 6A) and (ii) with the supernatants of monocytes previously cultured for 2 d with IL-26 (unpublished data), suggesting that T cell-monocyte contacts were dispensable. In the absence of CD3 triggering, IL-26 did not induce IL-17A secretion by memory T cells cultured with autologous monocytes (Figure 6A). These results suggest that IL-26 increases Th17 cell frequency by inducing the production of soluble mediators by monocytes.

IL-26 induces the secretion by monocytes of cytokines involved in human Th17 cell generation, such as IL-1-beta, IL-6, and TNF-alpha [31],[32]. We thus evaluated their potential implication in IL-26-induced IL-17A production. Memory CD4^+^ T cells were cultured with monocytes plus an anti-CD3 mAb and IL-26, in the presence of neutralizing anti-IL-1-beta, -IL-6, or -TNF-alpha mAbs, or of IL-1RA. IL-26-induced IL-17A secretion was inhibited by a neutralizing anti-IL-1-beta mAb and by IL-1RA (% inhibition = 87±6 and 79±12, respectively; mean ± SD, *n* = 5) but was unaffected by anti-IL-6 or -TNF-alpha mAbs (Figure 6B). IL-21 has been reported to favor naive T cell differentiation into Th17 cells [33]. IL-26-induced IL-17A secretion by memory CD4^+^ T cells was unaffected by a neutralizing anti-IL-21 mAb (unpublished data).

These results indicate that IL-26 increases the frequency of Th17 cells mainly through the induction of IL-1-beta production by monocytes.

### IL-26-Treated Monocytes Switch Memory CD4^+^ T Cells into Th17 Cells

We then evaluated whether IL-26 enhanced Th17 cell frequency by (i) increasing the proliferation of pre-existing Th17 cells and/or (ii) by polarizing non-Th17 committed memory T cells into Th17 cells.

**Figure 7:**
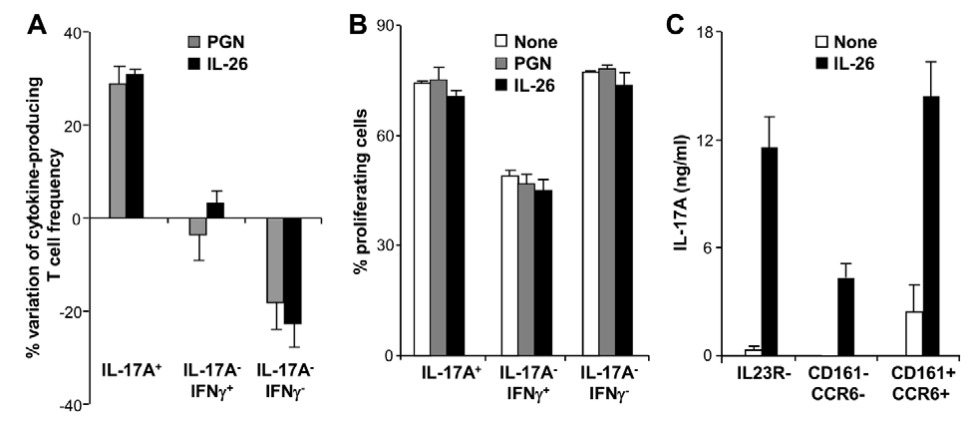
IL-26 induces the de novo generation of Th17 cells from memory CD4^+^ T cells. (A and B) Memory CD4^+^ T cell populations enriched in IL-17A-producing cells (>50%) were stimulated with an anti-CD3 mAb, in the presence of monocytes, with or without 50 ng/ml IL-26 or 5 µg/ml PGN. The frequency (measured at day 7; A) and proliferation (measured at day 5; B) of IL17A^+^, IL-17A^−^IFN-gamma^+^, and IL-17A^−^IFN-gamma^−^ T cells were analyzed by flow cytometry 6 h after stimulation with PMA plus ionomycin. Results are expressed as a percentage of variation of T cell frequency after stimulation, compared to untreated cells (A) or as a percentage of proliferating T cells (B) (mean ± SD, *n* = 4). (C) FACS-sorted IL-23R^−^, CCR6^−^ CD161^−^, and CCR6^+^ CD161^+^ memory CD4^+^ T cells were stimulated by an anti-CD3 mAb in the presence monocytes, with or without IL-26. IL-17A was quantified after 1 wk. Results are expressed in ng/ml (mean ± SD, *n* = 4).

Th17-enriched cell lines were stimulated with an anti-CD3 mAb and IL-26, in the presence of monocytes, and the frequency and proliferation of Th17 (IL-17A^+^ IFN-gamma^−^ and IL17A^+^ IFN-gamma^+^), Th1 (IFN-gamma^+^ IL-17A^−^), and IL-17A^−^ IFN-gamma^−^ T cell subsets were analyzed. IL-26 increased the frequency of IL-17A^+^ T cells and decreased the frequency of IL17A^−^ IFN-gamma^−^ (Figure 7A), without affecting their proliferation (Figure 7B). The frequency and proliferation of IFN-gamma^+^ IL-17A^−^ Th1 cells remained unaffected (Figure 7A and 7B). Consistent with these results, IL-26 did not modulate the proliferation (nor IL-17A secretion) of human Th17 cell clones stimulated by an anti-CD3 mAb and cultured with monocytes (unpublished data). Moreover, the neutralization of IL-23, a cytokine that favors Th17 survival and expansion rather than Th17 generation [32], did not affect IL-26-induced IL-17A secretion by memory CD4^+^ T cells (unpublished data). These observations suggest that IL-26 does not modulate the proliferation nor IL-17A production by established Th17 cells.

We therefore analyzed whether IL-26-stimulated monocytes may polarize IL-23R^−^ [22] and CCR6^−^ CD161^−^ [31],[34],[35] non-Th17 memory CD4^+^ T cells into Th17 cells. When stimulated with phorbol myristate acetate (PMA) plus ionomycin immediately after FACS sorting, these two populations did not produce detectable levels of IL-17A, as assessed by ELISA (unpublished data) and flow cytometry (Figure S2). Upon culture with IL-26-stimulated monocytes, IL-23R^−^ and CCR6^−^ CD161^−^ memory T cells acquired the ability to secrete IL-17A (Figure 7C), demonstrating that IL-26 favors Th17 cell generation by inducing non-Th17-committed memory T cell differentiation into Th17 cells. Moreover, IL-26 potentiated the production of IL-17A by CCR6^+^ CD161^+^ memory Th17 cells (Figure 7C).

These results show that IL-26 induces the differentiation of non-polarized memory T cells into Th17 cells.

### IL-26 in RA Fluids Triggers Proinflammatory Cytokine Production and Th17 Cell Generation

**Figure 8:**
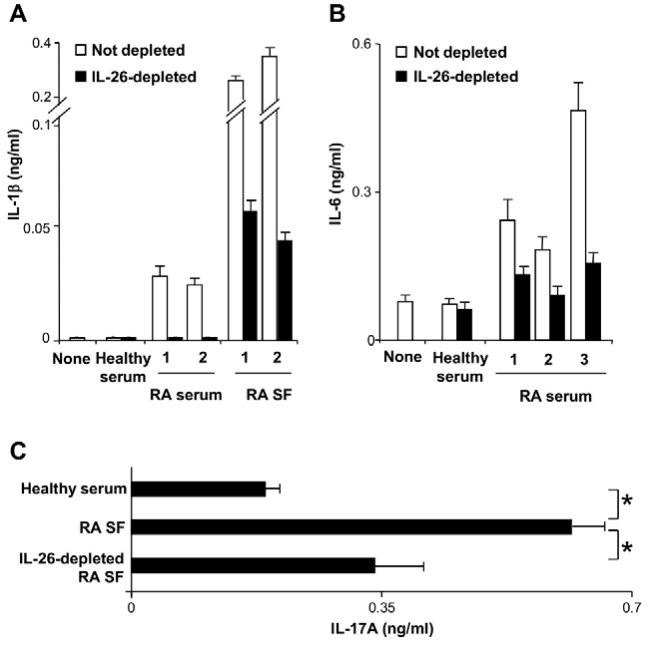
IL-26 present in the RA serums and SF is biologically active. (A and B) IL-1-beta (A) and IL-6 (B) were quantified by ELISA in the 48 h supernatants of monocytes (isolated from healthy subjects) cultured in X-VIVO-20 medium, supplemented with 10% human serum of three RA patients (which contained 10, 12, and 17 ng/ml of IL-26) or with 10% SFs of two RA patients (which contained 80 and 68 ng/ml of IL-26), either depleted or not in IL-26. Culture medium supplemented by 10% serum of RA patients or of healthy subjects contained less than 12 pg/ml IL-6, and IL-1-beta was undetectable in the culture medium supplemented by 10% SF and serums of RA patients. Experiments were performed with monocytes from four healthy subjects and results are expressed in ng/ml (mean ± SD, *n* = 4). For healthy subjects, the result is representative of one of four serums. (C) Memory CD4^+^ T cells were stimulated with an anti-CD3 mAb in the presence of monocytes, supplemented by 10% SF of three RA patients, depleted or not in IL-26, or with 10% serum of three healthy subjects, used as a control. IL-17A was quantified by ELISA in the 7-d supernatants. Results are expressed in ng/ml (mean ± SD, *n* = 3), and are representative of the results obtained with monocytes and T cells from one out of three healthy subjects. *p<0.05 (Wilcoxon matched-pairs signed-ranks test).

We next examined whether IL-26 present in RA fluids is biologically active. We first compared the ability of serums and SF of RA patients, either depleted or not in IL-26, to induce IL-1-beta and IL-6 secretion by monocytes. Monocytes produced higher levels of IL-1-beta (Figure 8A) and IL-6 when cultured with serums and SF from RA patients than with serums of healthy subjects (Figure 8B), while TNF-alpha remained undetectable (unpublished data). IL-26 depletion in RA serums and SF significantly reduced IL-1-beta and IL-6 induced by RA fluids (Figure 8A and 8B). The high concentrations of IL-6 in RA SF (5±1.8 ng/ml; mean ± SD; *n* = 9) avoided to evaluate the consequence of IL-26 depletion on IL-6 production.

IL-1-beta is a potent activator of monocytes present in RA fluids. We thus evaluated whether IL-26 and IL-1-beta in RA fluids may cooperate to stimulate monocytes. Neutralization of IL-1-beta in IL-26-depleted RA SFs decreased IL-6 mRNA expression in an additive manner (unpublished data), suggesting that IL-1-beta did not potentiate the effect of IL-26 on monocytes. Supporting these results, recombinant IL-1-beta did not potentiate recombinant IL-26-induced IL-6 and TNF-alpha production by monocytes (unpublished data).

Finally, we evaluated the capacity of IL-26 present in RA SF to induce Th17 cells. Memory CD4^+^ T cells were stimulated in the presence of RA SF, depleted or not in IL-26, or of serums of healthy subjects, used as controls. Compared to the serums of healthy subjects, RA SF enhanced IL-17A production by memory CD4^+^ T cells (% increase = 136±67; mean ± SD, *n* = 4) (Figure 8C). This effect was significantly reduced after IL-26 depletion (% decrease = 71±17%) (Figure 8C), but not totally prevented, supporting that other factors (such as IL-1-beta) may also favor Th17 cell generation. In contrast, the depletion of IL-26 did not affect IFN-gamma production (unpublished data).

Collectively, these results suggest that IL-26 in RA SF may promote locally Th17 cell generation.

## Discussion

RA is characterized by persistent synovial inflammation that leads to adjacent cartilage and bone destruction. TNF-alpha, IL-6, and IL-1-beta inhibitors are currently used to treat active RA, as these proinflammatory cytokines have a major role in the pathophysiology of RA [1],[2],[36]. However, to date, the factors that trigger proinflammatory cytokine production in RA remain unknown. We report here that IL-26 is overexpressed in RA and constitutively produced by synoviocytes in inflamed RA joints. IL-26 induces the secretion of proinflammatory cytokines (IL-1-beta, IL-6, and TNF-alpha) and chemokines (such as CCL20) by myeloid cells and favors the generation and local recruitment of Th17 cells. IL-26 thereby appears as a pivotal cytokine, located upstream of the local proinflammatory cascade, that may constitute a promising therapeutic target.

Through the secretion of IL-17A and proinflammatory cytokines, Th17 cells are critical players in the pathophysiology of RA [4]–[6] and other inflammatory disorders, such as inflammatory bowel disease, psoriasis, and allograft rejection [3],[31]. The identification of the factors involved in Th17 cell generation is thus crucial to design future therapeutic strategies to reduce IL-17A expression. We demonstrate here that IL-26 favors the generation of Th17 cells mainly through the induction of IL-1-beta production by monocytes. In addition to IL-1-beta, IL-21 plus TGF-beta have also been reported to induce human Th17 cell generation [30]–[33]. In our experiments, we have excluded the involvement of IL-21 and/or TGF-beta, on the basis of the following observations: (i) IL-26 does not induce IL-21 and TGF-beta expression by monocytes and T cells; (ii) a neutralizing anti-IL-21 mAb did not modulate IL-17A secretion by T cells cultured with IL-26-treated monocytes; and (iii) TGF-beta inhibited the IL-26-induced proinflammatory cytokine secretion by monocytes. Moreover, the generation of Th17 cells induced by IL-26 was not dependent on IL-23. IL-26 also induced the secretion of IL-6 and TNF-alpha by monocytes. However, and in agreement with others [32], we excluded a major role of TNF-alpha and IL-6 in human Th17 cell generation. In accordance with our results, IL-1-beta, in the presence of TNF-alpha and IL-6, has been shown to give rise to Th22 cells [37].

We observed that IL-26-stimulated monocytes induced the differentiation of non-Th17 committed (CCR6^−^ CD161^−^ and IL-23R^−^) memory T cells [31],[34],[35]. In contrast, IL-26 did not induce the polarization of naive T cells into Th17 cells. In agreement with this observation, only naive CD161^+^ CD4^+^ T cells present in post-natal thymus and umbilical cord blood have been reported to differentiate into Th17 cells [31],[34]. Different authors also failed to generate Th17 cells from circulating naive T cells isolated from adult subjects, even in the presence of IL-1-beta [31],[34],[35],[38].

**Figure 9:**
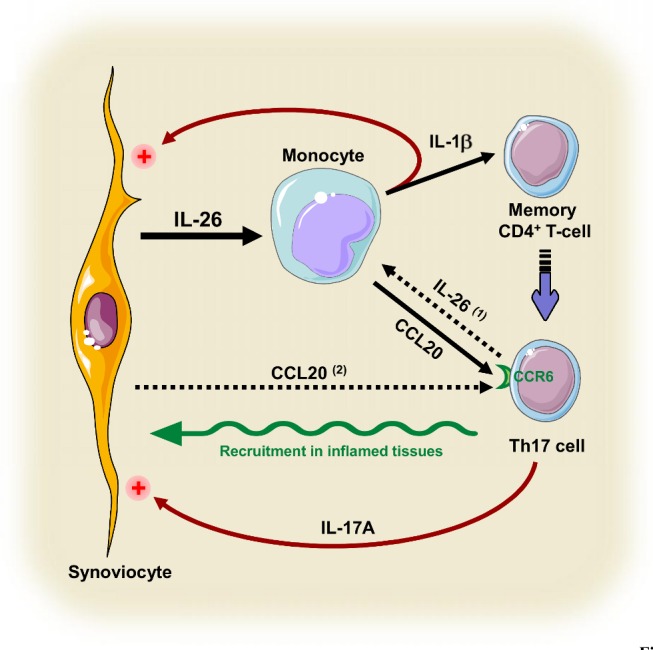
Schematic of the role of IL-26 in the Th17 cell polarization in RA. In inflamed RA joints, IL-26 is constitutively produced by synoviocytes and upregulates IL-1-beta and CCL20 secretion by myeloid cells. IL-1-beta favors the polarization of infiltrating CD4^+^ memory T cells into Th17 cells, and CCL20 promotes the recruitment of CCR6^+^ Th17 cells. In parallel, IL-1-beta and IL-17A, produced by monocytes and Th17 cells, respectively, increase IL-26 secretion by synoviocytes. IL-26 secreted by Th17 cells [1],[22] may also contribute to the production of proinflammatory cytokines by monocytes. Moreover, CCL20 produced by activated FLS may also contribute to local inflammation by inducing the recruitment of pathogenic Th17 cells in the synovium [2],[39]. This scheme was drawn using pictures from Servier Medical Art.

Interestingly, we observed that (i) IL-26 is expressed in the sublining layer of synovium and in T cell-rich areas, (ii) that myeloid cells infiltrating RA joints are sensitive to IL-26, and (iii) that IL-26 in RA fluids is biologically active and in sufficient concentrations to induce the production of proinflammatory cytokines by monocytes and of IL-17A by memory T cells. These results suggest that memory T cells, present in inflamed RA joints, could locally differentiate into Th17 cells, in the presence of IL-26 and infiltrating myeloid cells (as schematized in Figure 9). Through an upregulation of the expression of proinflammatory chemokines, and especially CCL20 [39], IL-26-exposed myeloid cells may also favor the local recruitment of activation of Th17 cells (Figure 9). Moreover, the expression of CCL20 by synoviocytes is increased by proinflammatory mediators, such as IL-1β and IL-17 [39], reinforcing the pivotal role played by FLS in the local recruitment of pathogenic Th17 cells (Figure 9).

RA synoviocytes constitutively express IL-26 and represent, with macrophages, the main source of IL-26 in RA joints. In addition to producing numerous factors involved in inflammation and matrix destruction [27], RA synoviocytes have been shown to migrate and to spread RA [27],[40],[41]. In a model of RA in SCID mice, RA FLS can migrate from an affected site into a distant healthy site, resulting in the subsequent cartilage invasion [40],[41]. The unabated secretion of IL-26 by RA synoviocytes may contribute to explain how they establish a proinflammatory microenvironment after migration into unaffected joints. Numerous proinflammatory factors (e.g., IL-1-beta and IL-17A) [27],[28] and some members of the IL-20 subfamily (such as IL-20 and IL-22) [42],[43] activate RA FLS. Interestingly, we report that IL-17A and IL-1-beta upregulate IL-26 secretion by RA FLS. This observation, added to the ability of IL-26 to upregulate IL-1-beta and IL-17A secretion by monocytes and T cells, respectively, evidence an amplification loop that may contribute to sustain the inflammatory process (Figure 9). Supporting observations in the intestinal lesions in Crohn's disease patients [21], we observed that some infiltrating T cells, including Th17 cells, express IL-26 in RA joints. It has been reported that IL-26 expression by Th17 cells depends on the stage of Th17 cell differentiation and on the local microenvironment [3]. This study may contribute to explain why IL-26 expression was detected only in some Th17 cells.

Despite the expression of the IL-26 heterodimeric receptor IL-10R2/IL-20R1 by RA (but not healthy) FLS [44], we failed in detecting an effect of IL-26 on RA and healthy FLS. Although the mechanisms responsible for the absence of cell sensitivity to IL-26 remain unknown, we could speculate that the constitutive production of IL-26 by RA FLS may promote the saturation/desensitization of the IL-26 receptor. Previous studies have also reported that primary epidermal keratinocytes [45] and HaCaT cells, after prolonged cultivation, express IL-10R2/IL-20R1 but do not respond to IL-26 (personal observations and [16]).

IL-26 was initially described as a cytokine that activates non-hematopoietic cells via the heterodimeric receptor IL-10R2/IL-20R1, inducing STAT1 and STAT3 phosphorylation [14]. We report that monocytes are sensitive to IL-26, in the absence of IL-20R1 expression ([19],[20] and personal observation). IL-26-induced monocyte activation (i) is reduced by a neutralizing anti-IL-10R2 mAb (Figure S3A) and (ii) is associated to IL-10R2 phosphorylation (Figure S3B), but not to STAT1 or STAT3 phosphorylation (personal observation). These results demonstrate that IL-26-induced signaling in monocytes involves IL-10R2, but not IL-20R1. Converging arguments support the expression by monocytes of an IL-26 receptor distinct from the heterodimeric receptor IL-10R2/IL-20R1, described on epithelial cells. First, supporting a model of IL-26/IL-20R1/IL-10R2 ternary complex structure described for monomeric IL-26 that does not apply for dimeric IL-26 [14], we observed that monomeric and dimeric IL-26 differ in their ability to activate epithelial cells and monocytes. While Colo205 cells are more sensitive to monomeric than dimeric IL-26 (Figure S4), monocytes are mainly sensitive to dimeric IL-26 (Figure 4H). In independent experiments, we observed that activated T and NK cells produce the dimeric form of IL-26 (personal observation). Second, although heparin interacts with IL-26 and abrogates IL-26-induced Colo205 activation (Figure S5A) [16], it dramatically potentiates monocyte activation (Figure S5B and S5C). Third, human B cells have been reported to respond to IL-26, in the absence of IL-20R1 expression [46]. Finally, IL-19, another member of the IL-20 cytokine subfamily that signals via IL-20R1/IL-20R2 [13], stimulates monocytes and T cells, despite the absence of IL-20R1 [47]. Currently, the nature of the IL-26 receptor expressed by human myeloid cells is under characterization.

The expression of IL-26 has been previously reported in the inflamed colonic lesions of patients with Crohn's disease [21] and in skin lesions of patients with psoriasis [22]. A role of IL-26 in local inflammation has also been suspected in a genome-wide association studies showing that SNP within the *il-26* gene region are susceptibility loci for inflammatory bowel disease with a high prevalence of articular involvement (i.e., RA, spondyloarthritis) [48],[49]. More recently, a meta-analysis identified the *il-26* gene-containing region as an ulcerative colitis risk locus [50]. In this study, we show that IL-26 is also overexpressed in RA patients and present at high concentrations in RA joints. These observations, added to the capacity of IL-26 to trigger inflammatory cytokine production by epithelial [21] and myeloid cells and to promote Th17 cell generation, suggest that IL-26 play an active role in the pathogenesis of chronic inflammatory diseases. In support, high levels of IL-26 are detected in arthritis other than RA, including osteoarthritis crystalline arthritis (personal observation). As IL-26 induces the secretion of active IL-1-beta (without affecting IL-1RA and IL-10 production), its involvement in sterile auto-inflammatory disorders (such as type II diabetes) should be investigated [51]. Finally, the beneficial interest of combining IL-26 blockade with IL-1-beta and/or TNF-alpha neutralization should be evaluated. In conclusion, the location of IL-26 upstream of the proinflammatory cascade highlights IL-26 as a novel and promising therapeutic target in RA and other chronic inflammatory disorders.

## Materials and Methods

### Patients and Healthy Subjects

This study includes 31 RA patients fulfilling the American College of Rheumatology criteria [52], 20 patients suffering from other inflammatory arthritis (ankylosing spondylitis, psoriatic arthritis, rhizomelic polyarthritis, and undifferentiated chronic inflammatory arthritis) (Table 1) and 26 healthy subjects. SFs of arthritis patients were collected at the time of joint effusion. Serums of healthy subjects were provided by the blood collection center (EFS Pays de la Loire, Angers, France). All samples were collected aseptically and centrifuged before storage at −20°C. This study has been performed in accordance with the guidelines of the ethics committee of the University Hospital of Angers (Angers, France).

**Table 1:** Demographic and clinical features of the patients.

Demographic and Clinical Features	RA	Other Inflammatory Arthritis
**Patients characteristics**		
Patients, *n*	31	20
Age, years	58.10±16.42	58.44±18.05
Sex ratio, M/F	8/23	10/10
*Disease duration, years*	*11±8*	*8.4±3.8*
ESR, mm/h	33.87±21.19	49.36±32.44
CRP, mg/dl	2.01±1.78	5.61±4.17
Rheumatoid factor, presence	22/31	0/20
Anti-CCP, presence	22/31	0/20
SF cellularity (cells/mm^3^)	11,180±7,388	9,463±7,420
Clinical score		
DAS28	5.10±1.27	
BASFI/BASDAI		30±16.37/25.67±9.29
**Medication at time of effusion**		
Prednisone	77.42%	45.00%
Methotrexate	67.74%	5.00%
Rituximab	6..45%	0.00%
Hydroxychloroquine	6.45%	0.00%
Sulfasalazine	3.23%	5.00%
Anti TNFa therapy	12.90%	0.00%
*NSAIDS*	25.81	30.00
No treatment	6.45%	0.00%
Anti TNFa therapy (previous)	19.35%	(-)
DMARDs (previous), *n*	83.87%	(-)

Values are represented in mean ± SD when relevant. Among 31 RA patients, 22 serums and 15 SF were collected. Other inflammatory arthritis include ankylosing spondylitis (*n* = 5; HLA B27 3/5 positive), rhizomelic pseudo-polyarthritis (*n* = 8), psoriatic arthritis (*n* = 4), and undifferentiated inflammatory arthritis (*n* = 3). Rhizomelic pseudo-polyarthritis, psoriactic arthritis, and undifferentiated inflammatory arthritis subjects are HLA-B27 −; anti CCP - and rheumatoid factor - Patients with infection and microcristallin arthritis were excluded from the study.

(-), not evaluated in the group; BASDAI, bath ankylosing spondylitis disease activity index; BASFI, bath ankylosing spondylitis functional index; CRP, C-reactive protein; DAS28, disease activity score using 28 joints counts; ESR, erythrocyte sedimentation rate; NR, not relevant; NSAID, non-steroidal anti-inflammatory drug; DMARD, disease-modifying anti-rheumatic drug.

### Antibodies

The origins and clone numbers of the mAbs used in this study are detailed in Table 2. The non-labeled and biotin-labeled goat anti-IL-26 polyclonal Abs were from R&D Systems. The rabbit anti-RORgamma t polyclonal Ab was from Abcam.

**Table 2:** List of the monoclonal antibodies used.

mAbs	Clone	Conjugate	Provider
Anti-CD3	UCHT1	FITC	BD Pharmingen
Anti-CD3	OKT3	None	ATCC
Anti-CD4	RPA-T4	APC or PE	BD Pharmingen
Anti-CD28	CD28.2	None	BD Pharmingen
Anti-CD45	HI30	Biotin	Invitrogen
Anti-CD68	KP1	FITC	Dako
Anti-CD161	DX12	APC	BD Pharmingen
Anti-CCR6	11A9	PE-Cy7	BD Pharmingen
Anti-IFN-gamma	B27	FITC	BD Pharmingen
Anti-IL-1beta	8516	None	R&D Systems
Anti-IL-6	1936	None	R&D Systems
Anti-IL-10R2	90220	None	R&D Systems
Anti-IL-17A	eBio64DEC17	Biotin or Alexa^647^	eBioscience
Anti-IL-17A	eBio64CAP17	PE	eBioscience
Anti-IL-22	142928	APC	R&D Systems
Anti-IL-23R	218213	PE	R&D Systems
Anti-IL-26	197505	None	R&D Systems
Anti-synoviolin	A-21	None	Santa Cruz biotechnology
Anti-TNF-alpha	1825	None	R&D Systems

### Fibroblast-like Synoviocytes

Primary FLS of RA patients and of healthy subjects were purchased from ECACC and cultured in synoviocyte growth medium (ECACC). 5×10^4^ FLS were stimulated or not with 50 ng/ml IL-1-beta (Milteny Biotech) and/or 50 ng/ml IL-17A (R&D Systems); IL-6 and IL-26 were quantified by ELISA in the 48 h supernatants.

### Cell Purification and Generation

Mononuclear cells were isolated from the peripheral blood mononuclear cells (PBMCs) of healthy donors and from RA SF, by density centrifugation using lymphocyte separation medium (PAA Laboratories). Then, T cell and non-T cell fractions were purified by positive or negative selection, respectively, using the CD3^+^ T cell isolation kit (Miltenyi Biotec), and were stimulated or not with 1 µg/ml anti-CD3 plus anti-CD28 mAbs or 10 µg/ml recombinant soluble CD40L (R&D Systems), respectively, in order to analyze IL-26 mRNA expression by reverse transcription (RT)-quantitative PCR (RT-qPCR) and IL-26 production (ELISA). T cell and non-T mononuclear cell purity, assessed by flow cytometry on a FACS Calibur (BD Biosciences), was >99% (unpublished data).

Naive (CD4^+^ CD45RA^+^ CD45RO^−^) and memory (CD4^+^ CD45RO^+^) T cells were purified from PBMC by negative selection (Miltenyi Biotec); purity, assessed by flow cytometry, was >99% (unpublished data). IL-23R^−^, CCR6^−^ CD161^−^, and CCR6^+^ CD161^+^ T cells were sorted from purified memory T cells with a FACS Aria (BD Biosciences) using PE-labeled anti-IL-23R mAb or PE-Cy7-labeled anti-CCR6 plus APC-labeled anti-CD161 mAbs; purity was >99% (Figure S2).

Monocytes from healthy subjects were isolated from PBMC by negative selection, using the EasySep human monocyte Enrichment kit (Stemcell). Cell purity, determined by flow cytometry, was routinely >95%. Monocytes were differentiated into macrophages or dendritic cells by culture at 1×10^6^ cells/ml for 5 d in complete medium (consisting of RPMI 1640 medium [Lonza] supplemented with 10% fetal calf serum [Biowest], 2 mM l-glutamine, 1 mM sodium pyruvate, 0.1 mM non-essential amino acids, 10 mM Hepes, 100 U/ml penicillin, and 100 µg/ml streptomycin [all from Lonza]) supplemented with 20 ng/ml GM-CSF or with 20 ng/ml GM-CSF plus 50 ng/ml IL-4 (both from Cellgenix), respectively. Circulating myeloid dendritic cells (mDCs) were isolated from PBMC using the CD1c^+^ (BDCA-1)^+^ Dendritic Cell Isolation kit (Miltenyi Biotec). Myeloid cells were isolated from RA SF mononuclear cells by CD14-positive selection (Miltenyi Biotec); purity assessed by flow cytometry analysis, was routinely >98%. In some experiments, synoviocytes were isolated from RA SF, as previously described [53].

### Myeloid Cell Stimulation

1×10^5^ monocytes from healthy subjects and CD14^+^ cells from RA patients were cultured (96-well flat bottom plate) in serum-free medium (X-VIVO-20; Lonza), containing 2 ng/ml GM-CSF, with or without homodimeric IL-26 (R&D Systems and eBioscience) at the indicated concentrations, or 5 µg/ml PGN from *Staphylococcus aureus* (Sigma-Aldrich). In some experiments, monocytes were cultured in the presence or absence of IL-26, used at the indicated concentrations, with or without (i) 20 ng/ml IL-4, IL-10, IL-13, TSLP, or TGF-beta (all from R&D systems); (ii) 10 µg/ml of a neutralizing anti-IL-26 mAb or an isotype control mAb; or (iii) 0.2 µg/ml polymixin B (Sigma-Aldrich); polymixin B activity was controlled by stimulating monocytes with 100 pg/ml LPS from the *E. coli* K12 strain (Cayla-InvivoGen). In other experiments, monocytes were cultured in the presence or absence of 50 ng/ml monomeric IL-26 (containing less than 10% homodimeric IL-26) (R&D Systems) or heat-treated (15 min at 95°C) homodimeric IL-26. Finally, monocytes were cultured with serums or SF of RA patients, depleted or not in IL-26, or with serums of healthy subjects. Depletion of IL-26 was performed by incubating RA serums or SF with mouse anti-IL-26 mAb for 12 h at 4°C, followed by incubation with protein A-sepharose beads (GE Healthcare) for 2 h at 37°C; controls consisted in incubating RA serums or SF with an IgG2b control mAb. Serums and SF were used at a final dilution of 10%, a concentration that did not affect cell viability (unpublished data).

### CD4^+^ T Cell Stimulation

1×10^5^ naive or memory CD4^+^ T cells, cultured (96-well U-bottom plate) in X-VIVO-20 medium, were stimulated with an immobilized anti-CD3 mAb, in the presence of 5×10^3^ autologous monocytes, with or without (i) IL-26, used at the indicated concentrations, (ii) RA serums or SF either depleted or not in IL-26, or (iii) 5 µg/ml PGN. In some experiments, 10 µg/ml neutralizing anti-IL-1-beta and/or anti-IL-6, anti-TNF-alpha, isotype control mAbs, or 1 µg/ml recombinant IL-1RA (R&D Systems) were added at the time of stimulation. IFN-gamma and IL-17A were quantified in the 7-d supernatants by ELISA. Results are expressed as concentrations or as percentages of inhibition of IL-17A production determined as follows: (A−B)/(A) * 100 were A and B correspond to the levels of IL-17A produced in the presence of IL-26 plus a control molecule and in the presence of IL-26 plus a neutralizing molecule, respectively. T cells were also cultured for one additional week with 20 ng/ml IL-23 (R&D Systems) and the frequency of IL-17A- and IFN-gamma producing T cells was determined by intracellular cytokine staining. In order to determine the role of T cell-monocyte contacts in the activation of T cells, 5×10^5^ memory CD4^+^ T cells and 2.5×10^4^ monocytes were cultured separately using 0.4-µm pore size Transwell insert (Corning Costar) in the presence of an anti-CD3 mAb, or 10^5^ T cells were cultured with 5×10^3^ monocytes in the absence of an anti-CD3 mAb. When CD4^+^ memory T cells were cultured without monocytes, 1 µg/ml anti-CD28 mAb was added, in the presence or absence of 10 ng/ml IL-1-beta and 50 ng/ml IL-6.

### Intracellular Cytokine Staining

After activation with 10 ng/ml PMA plus 1 nM ionomycin for 6 h in the presence of 10 µg/ml brefeldin-A (all from Sigma-Aldrich), T cells were fixed for 10 min at room temperature with PBS containing 4% paraformaldehyde (Euromedex). Intracellular cytokine staining was performed by incubating cells at room temperature for 30 min with 5 µg/ml FITC-labeled anti-IFN-gamma, 5 µg/ml PE-labeled anti-IL-17A, and 5 µg/ml APC-labeled anti-IL-22 mAbs in PBS containing 0.1% BSA and 0.1% saponin (both from Sigma-Aldrich). Staining was analyzed by flow cytometry with a FACS Calibur.

### Generation and Stimulation of Th17-Enriched Cell Populations

CD4^+^ memory T cells were enriched in IL-17A-secreting cells, as previously described [54]. Briefly, memory CD4^+^ T cells were stimulated with PMA plus ionomycin in the presence of anti-CD28 mAb for 3.5 h at 37°C. Cells were then incubated for 15 min in PBS containing 2% FCS, with biotin-labeled anti-CD45 mAb and biotin-labeled anti-IL-17A mAb, previously complexed with avidin (Invitrogen). Samples were then incubated with complete culture medium for 1.5 h at 37°C under agitation. Finally, cells were incubated with FITC-labeled anti-CD3, APC-labeled anti-CD4, and PE-labeled anti-IL-17A mAbs to sort IL-17A-producing CD4^+^ memory T cells using a FACS Aria. After standing for 48 h in RPMI 1640 medium supplemented with 8% human serum, cells were cultured for 15 d with 30 ng/ml IL-23, irradiated allogeneic PBMC, and EBV-B cells LAZ (gift from F. Jotereau, Inserm U892, Nantes, France). The percentage of IL-17A-secreting CD4^+^ memory T cells was determined by FACS and was >50% (unpublished data). CD4^+^ memory T cells enriched in IL-17A-producing cells were first labeled with the PKH26 labeling kit (Sigma-Aldrich) before stimulation for 5 d by anti-CD3 mAb in the presence of monocytes, without or with IL-26 or PGN. Then, cells were stimulated with PMA plus ionomycin in the presence of brefeldin-A for 6 h and incubated with Alexa^647^-labeled anti-IL-17A and FITC-labeled anti-IFN-gamma mAbs. Proliferation and cytokine expression were evaluated by flow cytometry using a FACS Calibur.

### Cytokine Quantification by ELISA

IL-26 was quantified by ELISA. Briefly, 96-well plates (Maxisorp; Nunc) were coated with 5 µg/ml polyclonal goat anti-IL-26 Ab (100 µl/well) (R&D Systems). After saturation with PBS containing 2% non-fat dry milk, plates were successively incubated with samples or recombinant human IL-26, with a biotinylated polyclonal goat anti-IL-26 Ab and then with streptavidin-HRP (BD Pharmingen). Plates were washed between each steps and bound Abs were revealed with the TMB substrate (Sigma-Aldrich). Optical density was measured at λ = 450 nm (Multiskan Ascent; Thermoelectron). The anti-IL-26 polyclonal Ab does not cross react with other IL-10 family members, as assessed by ELISA (Figure S6A). IFN-gamma, TNF-alpha, IL-1-beta, IL-4, IL-6, IL-10, IL-17A (all from Diaclone), IL-21 (eBioscience), and IL-22 were quiantified by ELISA (R&D Systems).

### Rheumatoid Factor Depletion

Rheumatoid factor was depleted by incubating RA fluids with anti-IgM Ab-agarose beads (Sigma-Aldrich) for 2 h at 37°C, followed by overnight incubation at 4°C. Depletion was verified by quantifying IgM by nephelometry (BN prospec).

### Quantitative RT-PCR Analysis

2×10^6^ monocytes were stimulated for 6 h with 50 ng/ml IL-26 in X-VIVO-20 medium. In other experiments, 2×10^6^ memory CD4^+^ T cells, cultured in X-VIVO-20 medium, were stimulated for 7 d with an immobilized anti-CD3 Ab, in the presence of 2×10^5^ autologous monocytes and 20 ng/ml IL-23, with or without 50 ng/ml IL-26. Memory CD4^+^ T lymphocytes were then sorted using CD4^+^ micro-bead kit (Miltenyi Biotec). Total RNA was extracted using Trizol and reverse transcribed using the superscript II RNaseH^−^ Reverse Transcriptase (both from Invitrogen). For qPCR, amplification was done using iQ SYBR Green Supermix (Bio-Rad) and specific gene expression was calculated using the 2^−ΔΔCT^ method (using GAPDH as calibrator). The primer sequences used are available upon request.

### Immunohistochemistry

After deparaffinization and antigen demasking, paraffin-embedded tissue slides of synovium from pathological joints of RA patients or of patients with recurrent dislocation were incubated with 10% human serum before labeling. For immunohistochemistry experiments, slides were labeled with an anti-IL-26 mAb or a control mAb. Bound mAbs were detected using EnVision+ Dual Link System-HRP (DAB+), following manufacturer's instructions (Dako). Slides were counterstained with hematoxylin and imaged with a ScanScope slide scanner (Aperio Corp). For immunohistofluorescence experiments, slides were incubated with biotinylated-anti-IL-26 mAb and -IgG2b control mAb, anti-RORgamma, anti-synoviolin, FITC-labeled-anti-CD68, FITC-labeled-anti-CD3, or FITC-labeled-isotype control mAb. Bound anti-IL-26 mAb and unlabeled mAbs were detected with Alexa Fluor 555-Streptavidin (Invitrogen) and FITC-labeled swine anti-rabbit IgG Ab (Dako), respectively. Slides were mounted in ProLongGold AntiFading reagent (Invitrogen) and imaged with a Nikon A1 R Si microscope (Nikon). The anti-IL-26 mAb does not cross react with other IL-10 family members, as assessed by Western-blotting analysis (Figure S6B).

### Western-Blotting Analysis

Recombinant dimeric and monomeric IL-26 were electrophoretically separated (50 ng/lane) on a 10% polyacrylamide gel in non-reducing conditions and then transferred to an Immobilon membrane (Millipore). After saturation, the membrane was incubated for 16 h at 4°C with 2 µg/ml anti-IL-26 mAb followed by incubation with 0.5 µg/ml peroxidase-labeled anti-mouse IgG Ab (Biosource). Bound Abs were detected using the ECL system, according to the manufacturer's instructions (Invitrogen).

### Statistical Analysis

Data were shown as mean ± SD and were analyzed by the Mann Whitney test or the Wilcoxon matched-pairs signed-rank test, depending on the experiment. Correlations between different variables were sought using Spearman's rank correlation and expressed as a rank correlation coefficient (*r*) and Pearson's correlation coefficient (p). *p*<0.05 was considered significant.

## Supporting Information

Figure S1 **Comparison of IL-26 and IL-1-beta levels in RA patients.** Correlations between IL-26 and IL-1-beta concentrations, quantified by ELISA, in the serums (*n* = 15; left panel) and SFs (*n* = 12; right panel) of RA patients, were analyzed using the Pearson's correlation test.
(TIF)

Figure S2 **Freshly purified IL-23R^−^, CCR6^−^ CD161^−^ memory T cells are devoid of Th17 cells.** (A) Purity of FACS-sorted T cells. IL-23R^−^, CCR6^+^ CD161^+^, and CCR6^−^ CD161^−^ memory T cells were isolated from peripheral blood T cells from healthy subjects by FACS. Purity was analyzed by FACS. (B) IL-23R^−^, CCR6^−^ CD161^−^, and CCR6^+^ CD161^+^ CD4^+^ memory T cells were FACS-sorted and the frequency of IL-17A and IFN-gamma producing cells were evaluated after 6 h stimulation with PMA plus ionomycin, in the presence of brefeldin A. (A and B) Results are representative of one out three independent experiments.
(TIF)

Figure S3 **The IL-10R2 receptor chain is involved in monocyte activation by IL-26.** (A) An anti-IL-10R2 Ab neutralizes IL-26-induced IL-6 production. Monocytes were cultured in X-VIVO-20 medium in the presence of 50 ng/ml IL-26, without or with 1, 5, or 10 µg/ml neutralizing goat anti-IL-10R2 Ab or isotype control Ab (both from R&D Systems). IL-6 was quantified in the 24 h supernatants. Results are expressed in percent of inhibition of IL-6 production (mean ± SD, *n* = 4) determined as follows: (A−B)/(A) * 100 where A and B correspond to IL-26-induced IL-6 production in the presence or the absence of the indicated concentrations of the Abs, respectively. (B) IL-26 induces IL-10R2 phosphorylation. Monocytes were stimulated or not with 50 ng/ml IL-26 or 50 ng/ml IL-10 for 15 min. After washing in cold PBS, cells were lysed and electrophoretically separated (50 ng/lane) on a phos-tag, according to the manufacturer's instructions (Wako chemicals), which provides a phosphate affinity SDS-PAGE for mobility shift detection of phosphorylated proteins. IL-10R2 phosphorylation was detected with a standard Western-blotting protocol using an anti-IL-10R2 Ab. Results are representative of one out three experiments.
(TIF)

Figure S4 **Monomeric IL-26 stimulates Colo205 cells more efficiently than dimeric IL-26.** Colo205 cells were cultured in CM, in the presence of different concentrations of monomeric or dimeric IL-26 (R&D Systems). IL-10 was quantified in the 48 h supernatants by ELISA. Results are expressed in pg/ml (mean ± SD; *n* = 4).
(TIF)

Figure S5 **Dual effect of heparin on IL-26-induced Colo205 cells and monocyte activation.** (A) Colo205 cells were cultured in CM with IL-26 at the indicated concentrations, with or without 10 µg/ml heparin. IL-10 was quantified by ELISA in the 24 h supernatants. (B) Monocytes were cultured in X-VIVO-20 medium in the presence of IL-26 as described, with or without 10 µg/ml heparin. IL-1-beta was quantified in the 48 h supernatants by ELISA. (C) Memory CD4^+^ T cells were stimulated by an anti-CD3 Ab in the presence of monocytes and 50 ng/ml IL-26, with or without 10 µg/ml heparin. IL-17A was quantified in the 7-d supernatants by ELISA. (A–C) Results are expressed in ng/ml (mean ± SD, *n* = 4).
(TIF)

Figure S6 **Analysis of anti-IL-26 antibody specificity.** (A) IL-10, IL-19, IL-20, IL-22, IL-24, and IL-26 (all from R&D Systems) were coated at 1 µg/ml (100 µl/well; Maxisorp 96-wells plate) and incubated with the goat anti-IL-26 Ab (1 µg/ml, 100 µl/well). After incubation with HRP-streptavidin, bound antibodies were detected with TMB substrate, followed by absorbance reading at 450 nm. Results are expressed in optical density values. (B) Western-blotting analysis of IL-10, IL-19, IL-20, IL-22, IL-24, IL-26, IL-28A, and IL-29 (50 ng/line; all from R&D Systems) recognition by the anti-IL-26 monoclonal Ab. (A and B) Results are representative of one out of three experiments.
(TIF)

## Abbreviations

FACS: fluorescence-activated cell sorting
FLS: fibroblast-like synoviocyte
IL: interleukin
IL-1RA: IL-1 receptor antagonist
LPS: lipopolysaccharide
mAb: monoclonal antibody
NK: natural killer
PBMC: peripheral blood mononuclear cell
PGN: peptidoglycan
PMA: phorbol myristate acetate
RA: rheumatoid arthritis
RT: reverse transcription
SD: standard deviation
TNF: tumor necrosis factor
