# Correction: IL-26 Is Overexpressed in Rheumatoid Arthritis and Induces Proinflammatory Cytokine Production and Th17 Cell Generation

**DOI:** 10.1371/annotation/22e63f1f-1a6e-4d53-8d33-06527d9a1dd4

**Published:** 2012-10-26

**Authors:** Murielle Corvaisier, Yves Delneste, Henry Jeanvoine, Laurence Preisser, Simon Blanchard, Erwan Garo, Emmanuel Hoppe, Benjamin Barré, Maurice Audran, Béatrice Bouvard, Jean-Paul Saint-André, Pascale Jeannin

The Author Summary section that follows the Abstract is included in the PDF, but is missing from the online version of the article. The text of the Author Summary is as follows.

Author Summary

Rheumatoid arthritis (RA) is a severe chronic inflammatory disease, characterized by joint inflammation and destruction. RA is characterized by immune responses involving the inflammatory T cell subpopulations Th1 and Th17. Therapeutic strategies currently aim to target inflammatory cytokines associated with these cells, the earlier-acting, the better. IL-26, a member of the IL-10 cytokine family, was reported overexpressed in Crohn’s disease, an autoimmune disease also involving Th1 cells. Curiously, the gene encoding IL-26 is absent in most rodents (including mice and rats). Here we evaluate the expression and role of IL-26 in RA using human cells. We have shown that IL-26 is constitutively expressed by synovial cells in RA joints. IL-26 induces the expression of other proinflammatory cytokines and chemotactic proteins by myeloid cells. Via the induction of IL-1-beta synthesis, IL-26 induces the differentiation of human memory T cells into Th17 cells. This study identifies IL-26 as a critical mediator, the activity of which is tissue restricted, located upstream of the inflammatory cascade, and involved in the generation of pathogenic Th17 cells. IL-26 is thus a promising therapeutic target to treat RA and other chronic inflammatory disorders.

