# Supplementary figures and images for: IL-26 Is Overexpressed in Rheumatoid Arthritis and Induces Proinflammatory Cytokine Production and Th17 Cell Generation
Source: PLoS Biol. 2012 Sep 25;10(9):e1001395. doi: 10.1371/journal.pbio.1001395 (PMC3463509; doi:10.1371/journal.pbio.1001395)

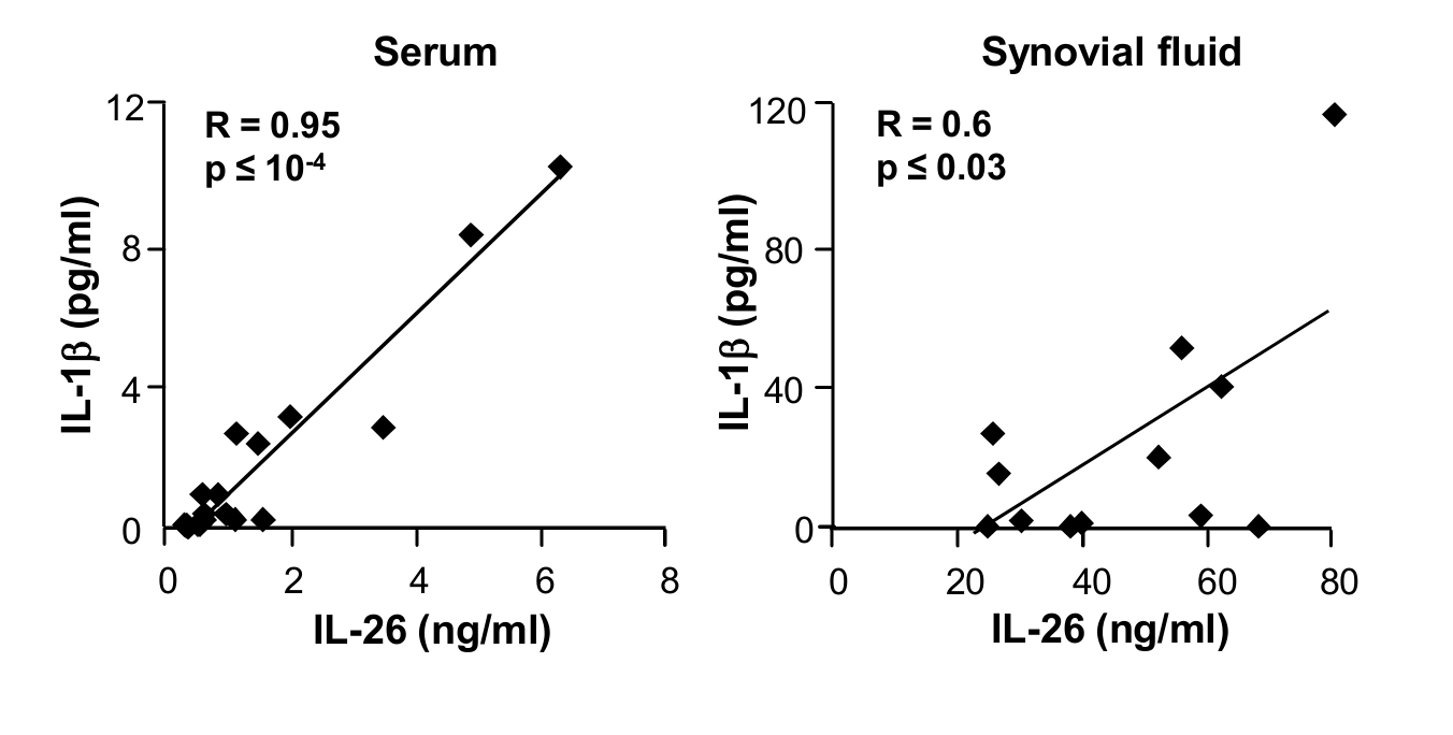

Supplement: Figure S1 — Comparison of IL-26 and IL-1-beta levels in RA patients. Correlations between IL-26 and IL-1-beta concentrations, quantified by ELISA, in the serums (n = 15; left panel) and SFs (n = 12; right panel) of RA patients, were analyzed using the Pearson's correlation test. (TIF) [file pbio.1001395.s001.tif]

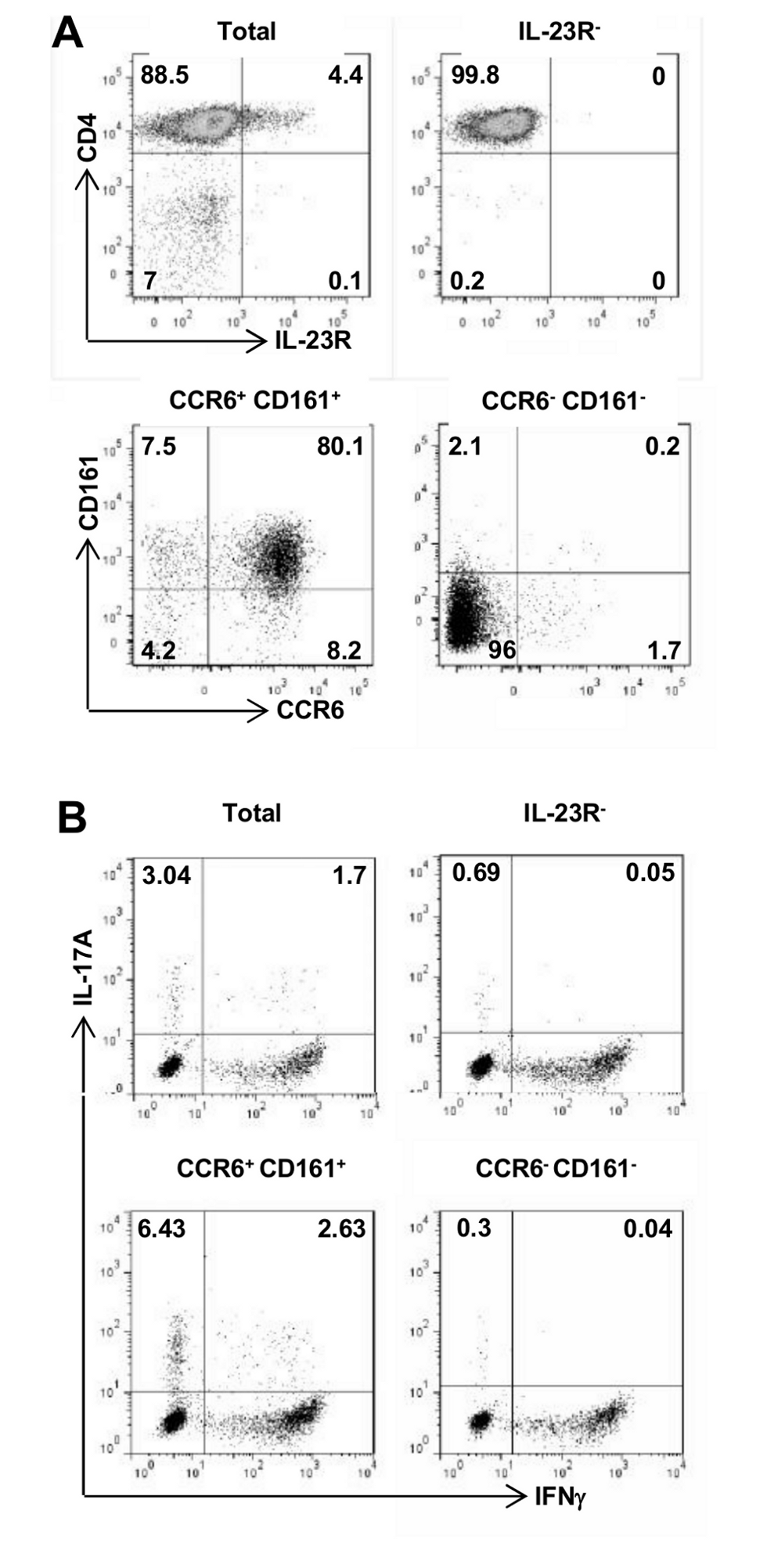

Supplement: Figure S2 — Freshly purified IL-23R−, CCR6− CD161− memory T cells are devoid of Th17 cells. (A) Purity of FACS-sorted T cells. IL-23R−, CCR6+ CD161+, and CCR6− CD161− memory T cells were isolated from peripheral blood T cells from healthy subjects by FACS. Purity was analyzed by FACS. (B) IL-23R−, CCR6− CD161−, and CCR6+ CD161+ CD4+ memory T cells were FACS-sorted and the frequency of IL-17A and IFN-gamma producing cells were evaluated after 6 h stimulation with PMA plus ionomycin, in the presence of brefeldin A. (A and B) Results are representative of one out three independent experiments. (TIF) [file pbio.1001395.s002.tif]

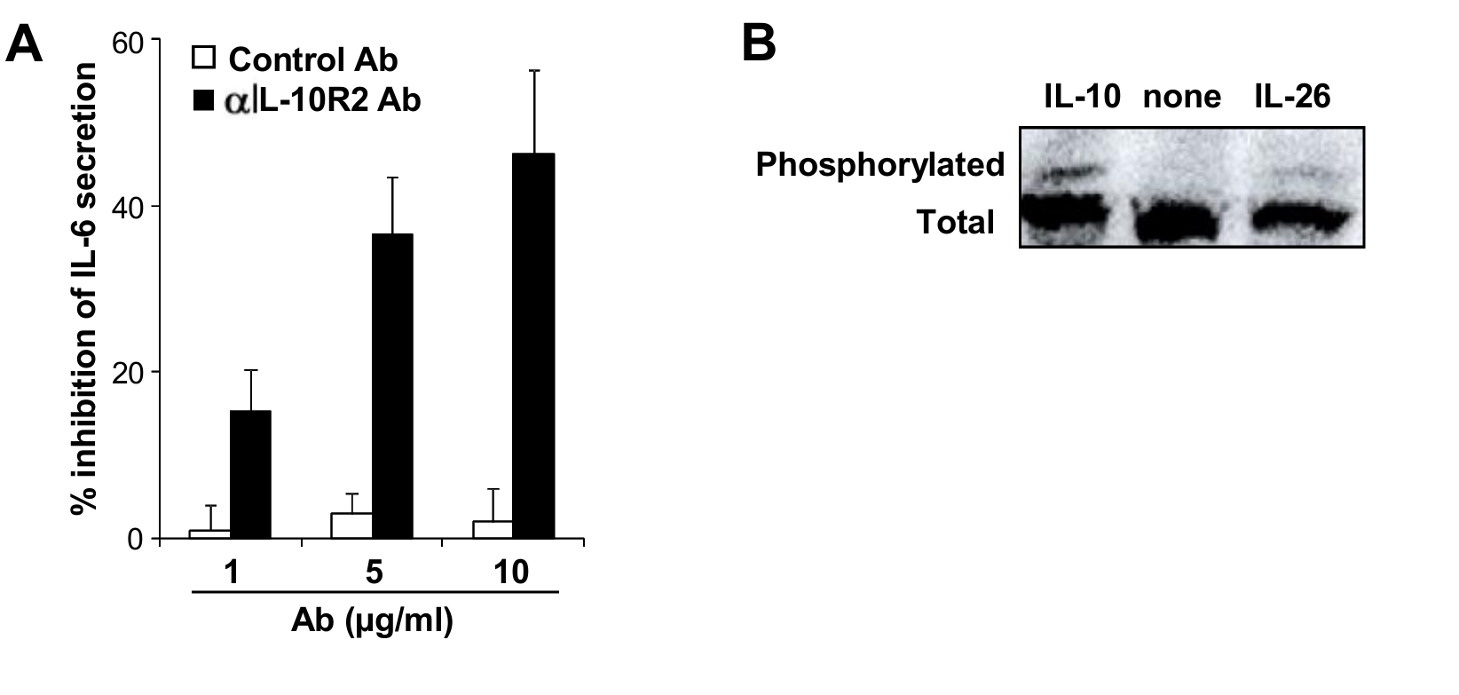

Supplement: Figure S3 — The IL-10R2 receptor chain is involved in monocyte activation by IL-26. (A) An anti-IL-10R2 Ab neutralizes IL-26-induced IL-6 production. Monocytes were cultured in X-VIVO-20 medium in the presence of 50 ng/ml IL-26, without or with 1, 5, or 10 µg/ml neutralizing goat anti-IL-10R2 Ab or isotype control Ab (both from R&D Systems). IL-6 was quantified in the 24 h supernatants. Results are expressed in percent of inhibition of IL-6 production (mean ± SD, n = 4) determined as follows: (A−B)/(A) * 100 where A and B correspond to IL-26-induced IL-6 production in the presence or the absence of the indicated concentrations of the Abs, respectively. (B) IL-26 induces IL-10R2 phosphorylation. Monocytes were stimulated or not with 50 ng/ml IL-26 or 50 ng/ml IL-10 for 15 min. After washing in cold PBS, cells were lysed and electrophoretically separated (50 ng/lane) on a phos-tag, according to the manufacturer's instructions (Wako chemicals), which provides a phosphate affinity SDS-PAGE for mobility shift detection of phosphorylated proteins. IL-10R2 phosphorylation was detected with a standard Western-blotting protocol using an anti-IL-10R2 Ab. Results are representative of one out three experiments. (TIF) [file pbio.1001395.s003.tif]

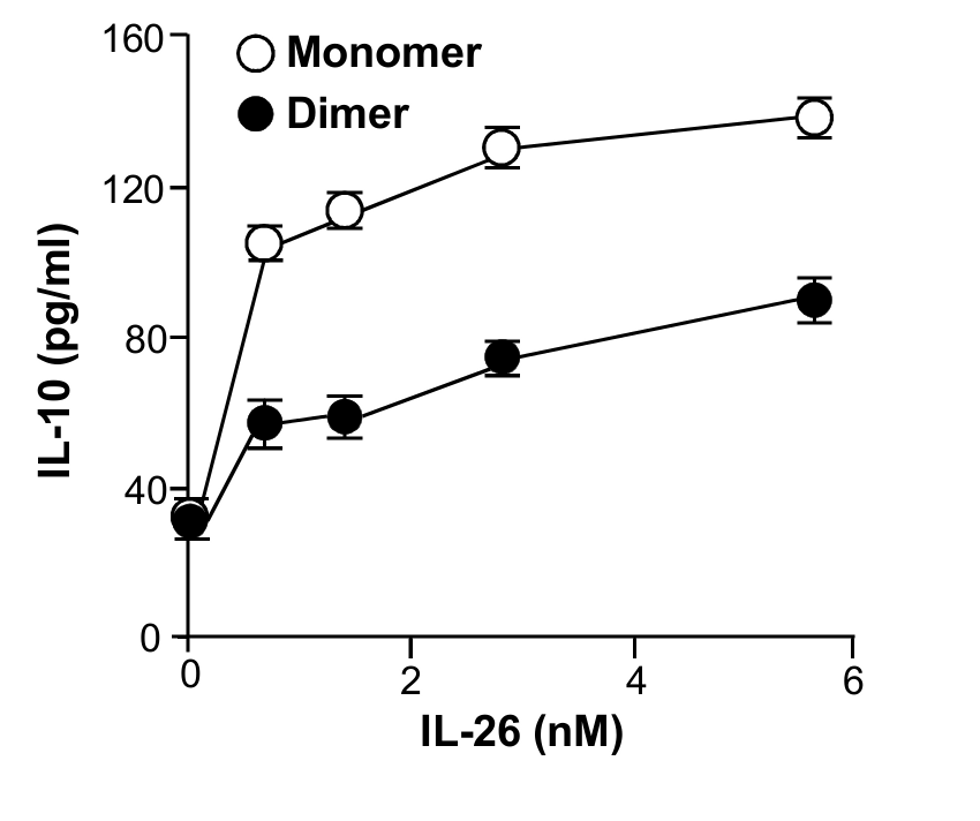

Supplement: Figure S4 — Monomeric IL-26 stimulates Colo205 cells more efficiently than dimeric IL-26. Colo205 cells were cultured in CM, in the presence of different concentrations of monomeric or dimeric IL-26 (R&D Systems). IL-10 was quantified in the 48 h supernatants by ELISA. Results are expressed in pg/ml (mean ± SD; n = 4). (TIF) [file pbio.1001395.s004.tif]

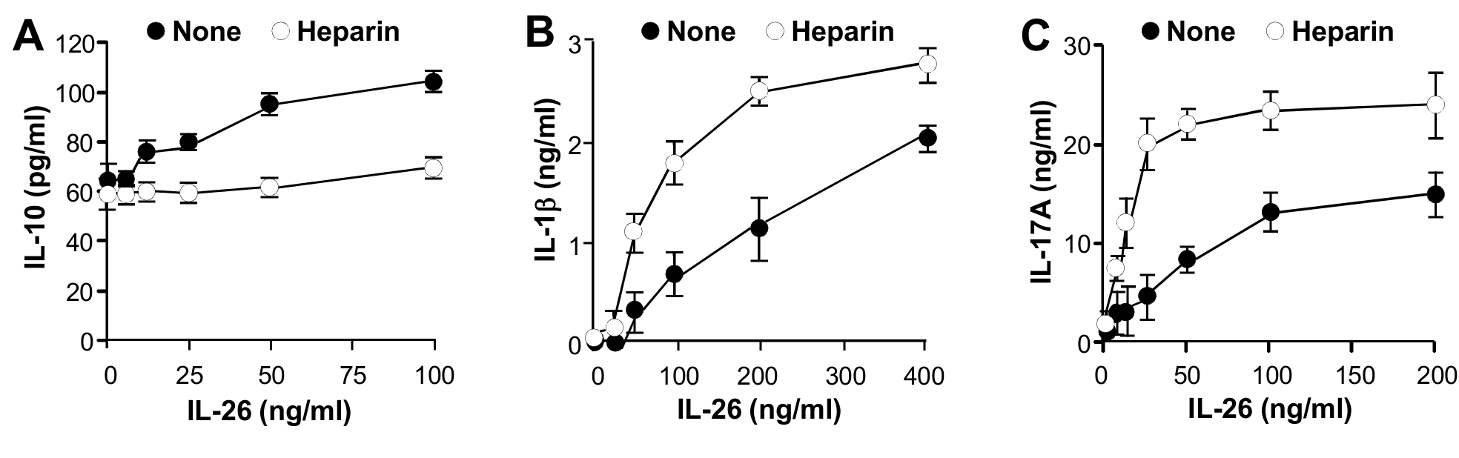

Supplement: Figure S5 — Dual effect of heparin on IL-26-induced Colo205 cells and monocyte activation. (A) Colo205 cells were cultured in CM with IL-26 at the indicated concentrations, with or without 10 µg/ml heparin. IL-10 was quantified by ELISA in the 24 h supernatants. (B) Monocytes were cultured in X-VIVO-20 medium in the presence of IL-26 as described, with or without 10 µg/ml heparin. IL-1-beta was quantified in the 48 h supernatants by ELISA. (C) Memory CD4+ T cells were stimulated by an anti-CD3 Ab in the presence of monocytes and 50 ng/ml IL-26, with or without 10 µg/ml heparin. IL-17A was quantified in the 7-d supernatants by ELISA. (A–C) Results are expressed in ng/ml (mean ± SD, n = 4). (TIF) [file pbio.1001395.s005.tif]

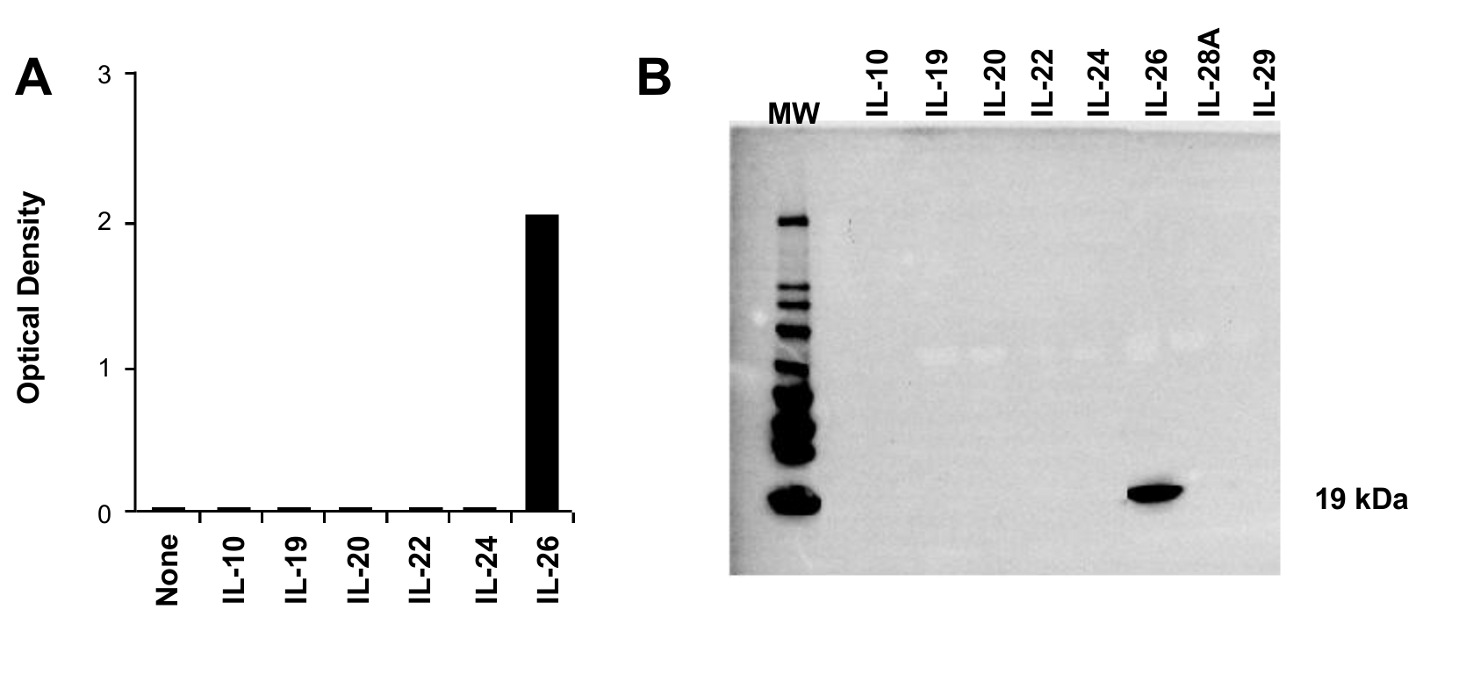

Supplement: Figure S6 — Analysis of anti-IL-26 antibody specificity. (A) IL-10, IL-19, IL-20, IL-22, IL-24, and IL-26 (all from R&D Systems) were coated at 1 µg/ml (100 µl/well; Maxisorp 96-wells plate) and incubated with the goat anti-IL-26 Ab (1 µg/ml, 100 µl/well). After incubation with HRP-streptavidin, bound antibodies were detected with TMB substrate, followed by absorbance reading at 450 nm. Results are expressed in optical density values. (B) Western-blotting analysis of IL-10, IL-19, IL-20, IL-22, IL-24, IL-26, IL-28A, and IL-29 (50 ng/line; all from R&D Systems) recognition by the anti-IL-26 monoclonal Ab. (A and B) Results are representative of one out of three experiments. (TIF) [file pbio.1001395.s006.tif]
